# Machine learning prediction models for mode of delivery in prolonged pregnancies in Sweden

**DOI:** 10.1038/s41598-025-19198-x

**Published:** 2025-09-12

**Authors:** Stefanie Schmauder, Anna Sandström, Magnus Boman, Christian Martin, Olof Stephansson

**Affiliations:** 1 Clinical Epidemiology Division, Department of Medicine Solna, Karolinska Institutet, Stockholm, Sweden; 2 Department of Obstetrics, Karolinska University Hospital, Stockholm, Sweden; 3 Division of Psychiatry, University College London, London, U.K.; 4 Center for Scalable Data Analytics and Artificial Intelligence (ScaDS.AI), Dresden/Leipzig, Germany

**Keywords:** Obstetrics, Machine learning, Register data, Late-term pregnancies, Induction of labour, Expectant management, Health care, Medical research

## Abstract

Induction of labour and not “expectant management” is often recommended to prevent adverse perinatal outcomes in late-term pregnancies, but no prognostic prediction model exists for an individualized decision-making. The present study used a data-driven approach to predict mode of delivery at or beyond 41 gestational weeks considering the obstetric management. Low-risk nulliparous women were derived from the nationwide Swedish Medical Birth Register (1998–2019). A two-day-wise prediction in four study groups with increasing gestational age (e.g. group 1: induced at 41^+0^-41^+1^, expectant management beyond 41^+1^ gestational weeks) was conducted. Forty-three features available at the time for decision-making on labour induction in each subgroup were used in the models, including the decision on labour induction itself. The subgroups contained 178,932, 129,449, 90,448 and 61,301 pregnancies, respectively, with imbalanced outcome rates (cesarean delivery < 27%, spontaneous birth > 55%, vaginal operative delivery < 18%). Five different classifiers were compared (random forest, mixed naïve bayes, support vector machine, neural network, logistic regression) with the highest value of the area under the curve being 69% in a hold-out sample. Although the considered features lacked predictive power, the study provides valuable methodological information for predicting the timing of labour induction beyond 41 gestational weeks.

**Supplementary Information:**

The online version contains supplementary material available at 10.1038/s41598-025-19198-x.

## Introduction

Induction of labour (IOL) is worldwide a common medical intervention to prevent adverse maternal and infant outcomes, especially after the estimated delivery date (≥ 40^+0^ gestational weeks (GW)). For example, the rates of one of the most devastating outcomes for a family, stillbirth, increase significantly beyond 40 GW^1^. The World Health Organization (WHO) recommends induction of labour for women who are known with certainty to have reached 41 GW^2^. A later onset of labour, “expectant management” (EM), should not be awaited. Recent systematic reviews with meta-analysis of randomised controlled trials (RCTs) have shown reduced risks of adverse fetal and neonatal outcomes (e.g. stillbirth, perinatal death) without an increase in maternal complications (e.g. cesarean section, vaginal operative delivery, postpartum hemorrhage) when IOL was performed at or beyond 37 GW compared to the respective EM^3,4^. Conducting IOL and not EM beyond 37 GW even seemed to be beneficial regarding cesarean section (RR 0.90 [95%CI: 0.85, 0.95])^3^. An individual participant data meta-analysis of RCTs found evidence that nulliparous but not multiparous women have a lower risk for an adverse composite perinatal outcome (perinatal mortality and severe perinatal morbidity) and for perinatal mortality (stillbirth and neonatal death) when induced at 41 weeks compared to EM until 42 weeks^5^. However, regarding other subgroups or outcomes, no sub-analysis could clearly identify the woman and child pairs who benefit the most from this intervention or narrow the time window at which IOL is indicated after 37 + 0 GW^3,5^. In contrast, the results of some observational studies, including propensity score matching, showed partly opposite results for selected perinatal outcomes^6–8^. A prospective cohort study alongside the INDEX RCT^9^, suggested a non-significant absolute risk reduction for adverse and severe adverse perinatal composite outcomes (including perinatal mortality and morbidity) and a higher risk for cesarean section in nulliparous women when IOL was performed at 41 + 0 compared to EM^6^. The women in the study were eligible for the trial but did not consent on randomisation and were treated according to their preferences. Studies with a propensity score matching design also indicated higher rates for cesarean section in this timeframe, even in multiparous women^7,8^.

Prognostic prediction models based on data-driven approaches could support the identification of pregnancies at risk for adverse outcomes and in the future provide more individualized advice for parents-to-be and medical staff. In recent years, several prediction models on adverse pregnancy- or birth-related outcomes were developed, of which some are already available for clinical use (e.g^10–16^. However, no model on outcomes in prolonged pregnancies exists. The present study therefore aims with its exploratory design to develop and internally validate a model which predicts mode of delivery (i.e. cesarean delivery (CD), vaginal operative delivery (VE) and spontaneous birth (SB)) considering induction of labour at different time thresholds of clinical relevance at or beyond 41^+0^ GW. For the report of the results we followed the TRIPOD + AI Expanded Checklist instructions for all sections of this publication^17,18^.

## Materials and methods

### Data

Data were derived from the Medical Birth Register (MBR) which covers around 98% of all births in Sweden and was established in 1973^19^. Data from ante-, intra- and perinatal care are collected prospectively by the National Board of Health and Welfare (NBHW)^19^. Through the unique personal identification number (PIN)^20^ data in the MBR is linked with the Cause of Death Register, the Patient Register for inpatient and outpatient specialized care (main and secondary diagnoses are coded according to the Swedish version of the ICD-system 10th revision since 1998^19^ and the Prescribed Drug Register (established 1952, 1964 and July 2005, respectively)^19–23^ at the NBHW.

The years included in the respective linked registers are summarized in Table S6 in the supplementary material. Table S7 in the supplement displays the register origin of each variable (outcomes and predictors) considered in the analysis^19^. Five used variables (mode of delivery, gestational length at birth, preeclampsia, preexisting chronic hypertension and preterm prelabour rupture of the membranes) have already been defined in previous studies^24–26^ based on existing variables in the MBR (Table S1, S8).”

### Participants

**Fig. 1:**
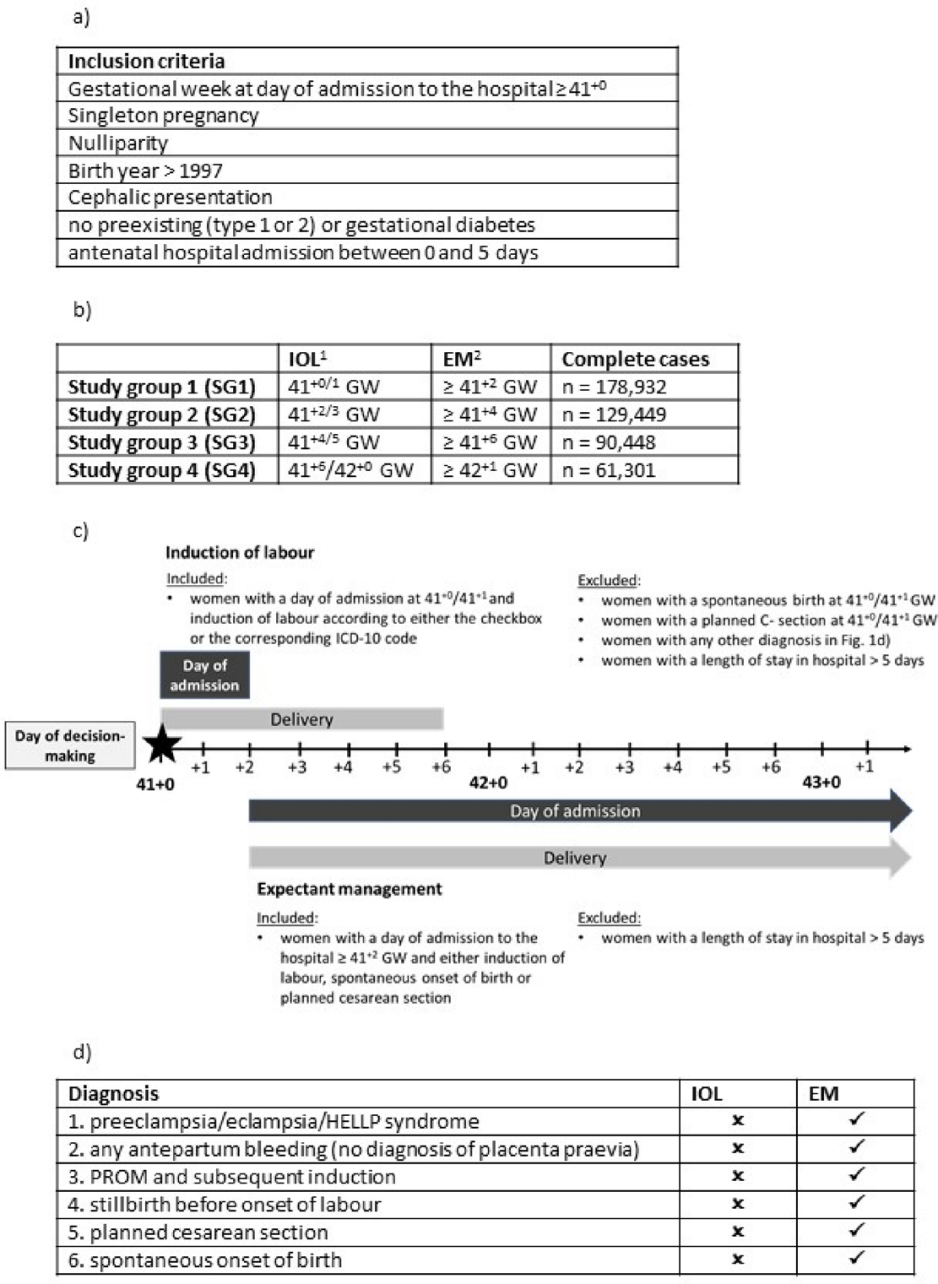
Design of the study population and the study groups 1-4. (**a**) Inclusion criteria applied to all registered pregnancies between 1992-2019. (**b**) Design of the study groups based on the study population. ^1^Induction of labour registered either through checkbox o corresponding ICD-10 code. The gestational age corresponds to the gestational age at the day of admission to the hospital. ^2^Later onsets of labour than the induced women in the respective group, including later IOL, planned cesarean and spontaneous birth. (**c**) Construction of study group 1 and the corresponding binary variable “induction of labour”. (**d**) 1.−4.: The most common diagnosis leading to an induction of labour (“confounding by indication”). Pregnancies with these diagnoses were excluded in the respective induction of labour group, while not in the respective expectant management group. Other onsets of labour (5 and 6) at the same gestational age as the induced women were excluded in respective study group. IOL: induction of labour. EM: expectant management. GW: gestational weeks. HELLP-Syndrom: acronym for hemolysis, elevated liver, low platelet counts. PROM: premature rupture of the membranes.

The initial study population consisted of nulliparous women across all registered age groups with a low-risk pregnancy who gave birth in Sweden at or beyond 41 + 0 GW between 1998 and 2019. Low-risk was defined as having a singleton fetus in cephalic presentation, no gestational or preexisting diabetes (Type 1 or 2) and an antenatal hospital admission not longer than five days before birth (Fig. 1a, Fig. S1 supplementary material). Pregnancies with known risk-factors are generally induced earlier than 41 + 0 GW.

We modified the inclusion criteria in the initial study population to reflect clinical decision-making and routines as well as for comparability to previous research from RCTs and observational studies^7–9,27,28^. Obstetricians and women usually decide during a consultation based on the medical findings if the pregnancy should be induced or not (referred to as day of decision-making). Study group 1 included women who were induced at 41^+0^- 41^+1^ (referred to as IOL) and women who delivered beyond 41^+1^ irrespective of onset of labour, i.e. elective cesarean delivery, IOL or spontaneous onset (referred to as EM). Women who delivered spontaneously, including premature rupture of the membranes (PROM) and subsequent induction, or by elective cesarean section at 41^+0^- 41^+1^ were excluded for Study group 1. In total, four different study groups (SG) were constructed, depending on gestational length and timing of IOL; SG1: IOL 41^+0^- 41^+1^ and EM > 41^+1^, SG2: IOL 41^+2^−41^+3^ and EM > 41^+3^, SG3: IOL 41^+4^−41^+5^ and EM > 41^+5^; SG4: IOL 41^+6^−42^+0^ and EM > 42^+0^ (Fig. 1b and c, Fig. S6 supplementary material).

The timing of IOL was calculated by using the gestational age at the day of admission to the hospital as a proxy (see section “Data preparation”) since the MBR does not provide the exact day of induction. Under clinical assumptions it is very unlikely that a woman at such a late stage of pregnancy (≥ 41 GW) is admitted to hospital without any intervention or giving birth (Fig. 1c, Fig. S6 supplementary material).

Women in each IOL group who had one of the following diagnoses registered were excluded to ensure that these women were only induced because of gestational age, not because of an underlying pathology: Hypertensive pregnancy disorders (Preeclampsia, eclampsia, HELLP-Syndrome), IUFD (intrauterine fetal death) or any antepartum bleeding (without a diagnosis of placenta previa) (Fig. 1d). Women with these diagnoses were not excluded in the corresponding EM group.

### Data preparation

The majority of variables are directly transferred to the NBHW from the standardized clinical records, where the information is generally collected through pre-specified checkboxes or through assigned diagnostic or procedure codes^19^. At the NBHW, the records are merged, quality checked, and annually released for the use as the MBR^19^.

The data-preprocessing steps conducted by the authors of the present study were to create the study population (see “Participants”) and the predictors. Variables for “gestational age at day of admission to the hospital”, “length of antenatal stay in hospital” (directly before birth), “onset of labour” and decision on IOL or EM (“induction of labour”) were created.

“Gestational age at the day of admission to the hospital” was calculated based on the existing variables “gestational age at delivery”, “birth date of the infant” and the “day of admission to the hospital”. The difference between the “birth date of the infant” and the “day of admission to the hospital” is the “length of antenatal stay in hospital” in days. In a second step the values for “length of antenatal stay in hospital” were subtracted from “gestational age at delivery” which resulted in the “gestational age at admission to the hospital”.

Onset of labour (elective cesarean, induction of labour or spontaneous onset) was classified hierarchically based on a checkbox for labour onset and a corresponding registered ICD-10 diagnosis (Table S1 Supplementary material). A premature rupture of the membranes with a subsequent induction of labour was classified as a spontaneous onset of labour (Table S1 supplementary material).

The binary variable “induction of labour” in the respective study group (SG1-SG4) was based on “gestational age at day of admission to the hospital” and “onset of labour”. Women classified as IOL in the respective group (Fig. 1b and c, Fig. S6 supplementary material) were the positive class and women who were classified as EM were the negative class.

Dimension reduction was done for smoking and snuff use. The three existing self-reported variables on smoking before, in early and late pregnancy with four categories (unknown, no smoking, 1–9 cigarettes/day, ≥ 10 cigarettes/day) were combined into two variables (smoking before and during pregnancy) with three categories (yes/no/unknown) (Table 1). Those, who smoked at any time during pregnancy were categorized as smoking during pregnancy.

**Table 1:** Descriptive analysis of the outcomes and included features in the four different study groups* (SG1-SG4).

	Study Group 1		Study Group 2		Study Group 3		Study Group 4	
	*n* = 197,567		*n* = 142,811		*n* = 99,714		*n* = 67,699	
Outcomes	n	%	n	%	n	%	n	%
Cesarean section	39,239	19.9	31,196	21.8	24,111	24.2	18,009	26.6
Vaginal operative delivery	33,457	16.9	24,601	17.2	17,377	17.4	11,876	17.5
Spontaneous vaginal birth	124,876	63.2	87,014	60.9	58,226	58.4	37,814	55.9
Categorical features								
Asthma	14,835	7.5	10,770	7.5	7,584	7.6	5,104	7.5
Antepartum Bleeding	842	0.4	595	0.4	393	0.4	206	0.3
Epilepsy	977	0.5	704	0.5	486	0.5	313	0.5
Antepartum PROM	168	< 0.1	117	< 0.1	81	< 0.1	58	< 0.1
IOL at the specific threshold of the respective study group	6457	3.3	5,310	3.7	4,679	4.7	9,918	14.7
Chronic hypertension	830	0.4	569	0.4	399	0.4	283	0.4
Nephrological disease	768	0.4	551	0.4	403	0.4	269	0.4
Assisted reproduction^1^	10,685	5.4	7,436	5.2	5,157	5.2	3,436	5.0
Systemic lupus erythematosus	156	< 0.1	102	< 0.1	64	< 0.1	36	< 0.1
Inflammatory bowel disease	1,216	0.6	841	0.6	597	0.6	387	0.6
Urinary tract infection	25,880	13.1	18,795	13.2	13,037	13.1	8,842	13.1
Smoking before pregnancy • unknown	35,642 17,682	18.0 8.9	25,840 12,702	18.1 8.9	18,197 8,745	18.2 8.8	12,571 5,912	18.6 8.7
Smoking during pregnancy • unknown	13,204 18,763	6.7 9.5	9,658 13,446	6.8 9.4	6,812 9,304	6.8 9.3	4,762 6,335	7.0 9.4
Snuff use before pregnancy • unknown	8,190 16,631	4.1 8.4	5,940 11,931	4.2 8.4	4,146 8,216	4.2 8.2	2,810 5,577	4.2 8.2
Snuff use during pregnancy • unknown	2,417 20,046	1.2 10.1	1,756 14,382	1.2 10.1	1,217 9,923	1.2 10.0	821 6,678	1.2 9.9
Mother’s country of birth								
• Sweden	159,379	80.7	115,372	80.8	80,422	80.7	54,391	80.3
• Other Nordic	2,899	1.5	2,109	1.5	1,488	1.5	1,007	1.5
• Europe	11,718	5.9	8,339	5.8	5,796	5.8	3,963	5.9
• Other	23,306	11.8	16,803	11.8	11,869	11.9	8,246	12.2
• Missing	265	0.1	188	0.1	139	0.1	92	0.1
Father’s citizenship								
• Sweden	165,790	83.9	120,059	84.1	83,859	84.1	56,837	84.0
• Other Nordic	2,538	1.3	1,837	1.3	1,296	1.3	876	1.3
• Europe	7,052	3.6	5,055	3.5	3,469	3.5	2,355	3.5
• Other	13,709	6.9	9,813	6.9	6,859	6.8	4,772	7.0
• Missing	8,478	4.3	6,047	4.2	4,231	4.2	2,859	4.2
Family situation								
• living with the infant’s father	173,720	87,9	125,698	88.0	87,867	88.1	59,725	88.2
• Single	15,111	7,6	11,024	7.7	7,756	7.8	5,276	7.8
• Other	8,736	4,4	6,089	4.3	4,091	4.1	2,698	4.0
Numerical features	mean (std)		mean (std)		mean (std)		mean (std)	
Antenatal visits • missing	11.3 (3.2) *n* = 6,760 (3.4%)		11.4 (3.2) *n* = 4,714 (3.3%)		11.5 (3.2) *n* = 3,156 (3.2%)		11.6 (3.3) *n* = 2,114 (3.1%)	
Maternal age at delivery (years) • missing	28.8 (13–52)^4^ *n* = 2 (0.0%)		28.8 (13–52)^4^ *n* = 2		28.8 (13–52)^4^ *n* = 2		28.9 (13–52)^4^ *n* = 2	
Mothers height (cm) • missing	167.0 (6.4) *n* = 9,167 (4.6%)		167.1 (6.4) *n* = 6,490 (4.5%)		167.0 (6.4) *n* = 4,394 (4.4%)		167.0 (6.5) *n* = 2,961 (4.4%)	
Mother’s weight (first antenatal visit) (kg) • missing	68.8 (13.5) *n* = 14,825 (7.5%)		69.0 (13.6) *n* = 10,575 (7.4%)		69.3 (13.7) *n* = 7,296 (7.3%)		69.5 (13.9) *n* = 5,001 (7.4%)	
Early pregnancy BMI (kg/m^2^) ^2^ • missing	24.6 (4.5) *n* = 15,452 (7.8%)		24.7 (4.6) 11.044 (7.7%)		24.8 (4.6) *n* = 7,632 (7.7%)		24.9 (4.6) *n* = 5.221 (7.7%)	
Number of previous spontaneous miscarriages^3^	1.3 (0.6) (*n* = 29,254)		1.3 (0.6) (*n* = 21.002)		1.3 (0.6) *n* = 14,771		1.3 (0.6) *n* = 10,113	
Involuntary childlessness (years)^3^	2.6 (2.0) (*n* = 23,527)		2.6 (2.0) (*n* = 16,773)		2.6 (2.0) *n* = 11,773		2.6 (2.0) *n* = 7969	

^1^any method (specify), ^2^imputed with mother’s height, ^3^self-reported, ^4^ mean (minimum-maximum).

* SG1: induction of labour (IOL) at 41 + 0–41 + 1 and expectant management (EM) > 41 + 1, SG2: IOL at 41 + 2–41 + 3 and EM > 41 + 3, SG3: IOL 41 + 4–41 + 5 and EM > 41 + 5; SG4: IOL 41 + 6–42 + 0 and EM > 42 + 0.

### Outcomes

The main outcomes of the study were mode of delivery categorized into cesarean section, vaginal operative delivery (forceps or ventous) and spontaneous vaginal birth. Each outcome was predicted separately (no composite outcome).

### Predictors

To ensure a predictive design with a potential for prospective use, only variables which are known at the day of decision-making regarding IOL in each study group (Fig. 1c) were used as features (Table 1). These features included diagnoses of some diseases which did not meet the exclusion criteria of the study (asthma, antepartum bleeding, epilepsy, chronic hypertension, nephrological disease, systemic lupus erythematosus, inflammatory bowel disease, urinary tract infection), as well as pregnancy-related variables (decision on IOL or EM, antepartum PROM, assisted reproduction, number of antenatal visits in antenatal care, self-reported number of previous spontaneous miscarriages, number of years of involuntary childlessness, smoking/snuff use before or during pregnancy, and mother’s height, weight and BMI at first antenatal visit) and sociodemographic predictors (mother’s country of birth, father’s citizenship, family situation). All non-binary categorical variables were one-hot encoded resulting in *n* = 43 features used in the prediction models. Diagnoses and clinical characteristics which occurred later (i.e. during expectant management) or during labour were not considered.

### Missing data

Missing values for the included variables range over time but rarely exceeds 5–10% with a further decreasing trend since the start of the digitalized report to the NBHW in 2007^19,29^.

In the present study population missing values did not exceed 5% beside body mass index (BMI) and smoking/snuff use (Table 1, Table S2 supplementary material). Missing values were treated according to the mechanism why they were missing (e.g. missing completely at random, missing at random, missing not at random). We classified BMI as missing not at random. It cannot be ruled out that mother’s pre-pregnancy BMI is calculated only when overweight or obesity is obvious^30^, as well as technical issues^29^. The rate of missing values of maternal pre-pregnancy height are below 5% (Table 1) and the validity is considered high^19^. However, the rate of missing values for BMI before imputation in the four study groups ranged between 8.3 and 8.5% (data not shown). BMI was imputed by using mother’s height from a next pregnancy registered in the MBR during the included time-period, which decreased the rate of missingness to 7.7–7.8% (Table 1.)

We also considered the variables smoking and snuff use to be missing not at random. Although the validity of the information on smoking is high, there is evidence on relevant underreporting of active smoking in early and late pregnancy by self-reported quitters^31^. Assuming that women start smoking (using snuff) during pregnancy very rarely^29^, missing values were replaced based on the values registered at other time points, i.e. smoking before, in early or late pregnancy (Table S2 supplementary material). The remaining missing values (e.g. values for smoking before pregnancy were not registered before 1999) were summarized in the category “unknown” (Table 1).

Introducing a category “unknown” for categorical variables reflects the nature of register data as well as the clinical setting and is in line with the literature^11^. This was also applied for the variables for mother’s country of birth and father’s citizenship (Table 1).

An exception in the registers are the diagnoses which are registered by checkboxes. In these cases, there is only a registered value, if the respective diagnosis is present. No missing values can be calculated. The usual procedure for variables based on checkboxes is to replace missing values with zero (no event occurred).

Another exception are the variables “number of previous spontaneous abortions” and “years of involuntary childlessness”, which are self-reported. Zero values (no event occurred) are not registered. For descriptive analysis the missing values were not considered and the mean was only calculated for those women who reported at least one event (Table 1). For the machine learning analyses, the missing values were replaced by zero, meaning that no such event occurred. Similar to the checkboxes, no amount of missingness could be calculated.

For the creation of the study population, observations with missing values in some of the variables had to be excluded. However, missing values for the respective variables did not exceed 1,6% (Fig. S1 supplementary material).

### Analytical methods

A complete case analysis was conducted in all models. Observations with at least one missing value in the outcome or predictor variables were excluded (Table 1, Fig. S1-S5 supplementary material). Data preprocessing and handling of missing values were the same for all analyses in each study group.

In each study group, five different classifiers (random forest, support vector machine, neural network, mixed naïve bayes and logistic regression) were compared while predicting every chosen outcome in a binary classification. According to the requirements of the respective classifier, continuous variables were standardized with standard scaler (zero mean and unit variance) for logistic regression, support vector machine and neural network.

Additionally, a multiclass classification analysis was conducted using the best-performing classifier from the separate binary outcome evaluations, in order to account for potential inconsistencies or overlaps in the one-vs-rest approach and to assess performance for the mutually exclusive modes of delivery. This approach ensures a single, unambiguous prediction per observation, which better reflects the clinical decision-making task.

The four study groups were randomly split into a training (70%), a validation (10%), and a test (20%) set with stratification for the outcome rate in each analysis (Table S3 supplementary material). Because of the exploratory approach, initially non-tuned analyses with the default values of each classifier were run and presented to have a benchmark comparison.

All steps of the analyses including data-preparation were processed with Python Version 3.10. The code was written using the scikit-learn machine learning library for the support vector machine (linear kernel), random forest (n_estimators = 100, max_depth = None, min_split = 2 as default values in the library) and logistic regression model (penalty = None). TensorFlow open source machine learning framework was applied to build the neural network. The model was constructed with two hidden layers (64 and 32 neurons respectively) using ReLU activation function and one output layer with a single neuron and a sigmoid activation function for binary classifications. After configuration (optimizer = ‘adam’, loss = ‘binary_crossentropy’) the model was trained for 10 epochs. For the multiclass prediction we used the cross entropy softmax activation function (sparse_categorical_crossentropy) in the output layer, appropriate for mutually exclusive classification tasks. All other characteristics of the neural network architecture and training procedure remained the same as in the binary classification setting. We used the mixed-naïve-bayes package for categorical and Gaussian Naïve Bayes^32^.

### Performance metrics

The performance of the models was evaluated by plotting receiver operating characteristic curves (ROC) and precision recall curves (PR) for each outcome in each group. To quantify the performance, the corresponding areas under the curves (auROC, auPR) were calculated with the roc_auc_score function and average_precision_score from scikit-learn. The respective 95% confidence interval (CI) was assessed by applying the RepeatKFold function with 5 splits and 20 repeats (scikit-learn library).

While auROC is measuring how well the respective algorithm is able to distinguish between the positive and the negative class across different thresholds, auPR provides additional information on performance in case of imbalanced data sets. The auPR is a metric for evaluating the prediction of the positive class only.

Calibration curves were additionally plotted to analyze the reliability of the predicted probabilities for each outcome. The curves were plotted by using CalibrationDisplay.from_predictions (scikit-learn library). The package bins the predicted probabilities into n numbers of bins and calculates both the mean predicted probability and the fraction of true positives in each bin. We chose *n* = 10 bins to depict every 10% of predicted probabilities. To provide more information of the distribution of the predicted probabilities, histograms were plotted which map how often the respective probabilities were predicted throughout the range (0% − 100%).

Sensitivity, specificity, positive predictive value (PPV) and balanced accuracy were calculated at a 50% threshold (default value).

For consistency, the additional multiclass prediction model was evaluated by calculating the same performance metrices as described for the binary outcomes (sensitivity, specificity, PPV, balanced accuracy, auROC, auPR and calibration curves including histograms for predicted probability).

For the support vector machine classifier, performance metrics on a 50% threshold and not auROC or auPR were evaluated as this classifier does not directly provide probability estimates. The values summarized in the classification report were very low (Table S4 supplementary material).

## Results

### Study population and study groups

The initial study population comprised 260,718 healthy nulliparous women with a singleton pregnancy in cephalic presentation, a date of admission to the hospital at or beyond 41 + 0 GW and an antenatal stay ≤ 5 days (Fig. S1 supplementary material). The four study groups derived from the population included 197,567 (SG1), 142,811 (SG2), 99,714 (SG3) and 67,699 (SG4) pregnancies, respectively (Fig. S1-S5 supplementary material). The outcome rates were imbalanced and differed between the groups with a trend towards an increase in cesarean Sect. (19.9% (SG1) – 26.6% (SG4)) and vaginal operative delivery (16.9% (SG1) – 17.5% (SG4)), and towards a decrease for spontaneous vaginal birth (63.2% (SG1) – 55.9% (SG4)) (Table 1). The distribution in the selected features were comparable in all study groups (Table 1).

After removing observations with at least one missing value the study groups consisted of 178,932 (SG1), 129,449 (SG2), 90,448 (SG3) and 61,301 complete cases (Fig. 1b).

### Model performance

**Fig. 2:**
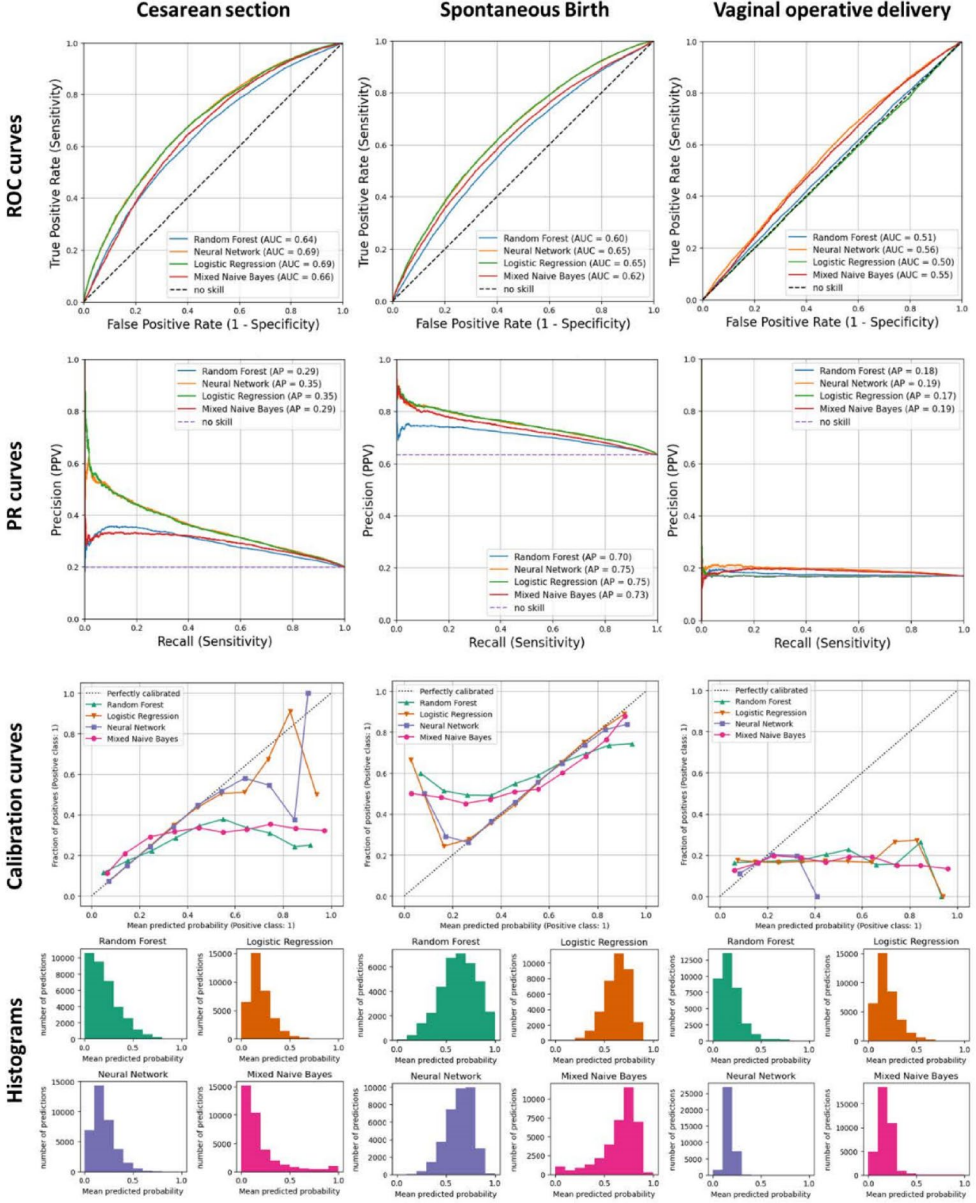
Performance metrics (ROC Curves, PR Curves, Calibration Curves) of the models in study group 1 (SG1). ROC curve: receiver operating characteristic curve; PR curve: Precision-Recall Curve; AUC: area under the curve.

In SG1, logistic regression and neural network had the highest auROC with 69% in predicting cesarean section and 65% in predicting spontaneous vaginal birth. The auROC for vaginal operative delivery reached a maximum of 56% with the neural network, while logistic regression predicted not better than chance (Table 2; Fig. 2).

**Table 2:** Performance metrics of the different models in study group 1 for all outcomes.

		CD	SB	VE**
LR	Sensitivity	6%	90%	0%
	Specificity	99%	24%	100%
	Precision	53%	67%	0%
	Balanced accuracy	52%	57%	50%
	auROC (95%CI)	68.70% (68.69–68.70)	65.15% (65.14–65.15)	50%
	auPR (95%CI)	35.38% (35.37–35.38)	74.72% (74.71–74.72)	17%
MNB	Sensitivity	15%	87%	1%
	Specificity	92%	23%	99%
	Precision	33%	66%	17%
	Balanced accuracy	54%	55%	50%
	auROC (95%CI)	65.84% (65.83–65.85)	62.38% (62.36–62.40)	55%
	auPR (95%CI)	29.17% (29.16–29.18)	73.02% (73.00-73.04)	19%
NN	Sensitivity	5%	91%	0%
	Specificity	99%	22%	100%
	Precision	53%	67%	0%
	Balanced accuracy	52%	57%	50%
	auROC (95%CI)	68.77% (68.75–68.79)	64.84% (64.82–64.86)	56%
	auPR (95%CI)	35.25% (35.21–35.28)	74.38% (74.36–74.40)	19%
RF	Sensitivity	10%	80%	2%
	Specificity	96%	33%	98%
	Precision	36%	67%	20%
	Balanced accuracy	53%	56%	50%
	auROC (95%CI)	64.32% (64.29–64.35)	60.16% (60.13–60.18)	51%
	auPR (95%CI)	29.02% (28.99–29.04)	70.40% (70.37–70.43)	18%
SVM*	Sensitivity	1%	100%	0%
	Specificity	100%	1%	100%
	Precision	56%	63%	0%
	Balanced accuracy	51%	50%	50%

SG1: induction of labour (IOL) at 41 + 0–41 + 1 and expectant management (EM) > 41 + 1. CD: Cesarean delivery, SB: spontaneous birth, VE: Vaginal operative delivery. Sensitivity, specificity and precision are calculated on a 50% threshold. LR: logistic regression, MNB: mixed naïve bayes, NN: neural network, RF: random forest, SVM: support vector machine. auROC: area under the receiver operating characteristic curve, auPR: area under the precision-recall curve. 95%CI: 95% confidence interval. * no ROC curves or PR curves were calculated for SVM. ** no confidence intervals were calculated for auROC and auPR in VE.

The auPR differed between the outcomes with reaching 75% for spontaneous vaginal birth and 36% in cesarean section (Table 2; Fig. 2). Logistic regression and neural network models again performed better than mixed naïve bayes and random forest models. The auPR for vaginal operative delivery was below 20% (Table 2; Fig. 2).

The neural network and the logistic regression model were almost perfectly calibrated for the prediction of spontaneous vaginal birth and well calibrated for the prediction of cesarean section below 60% of predicted probabilities (Fig. 2, Calibration curves). Random forest and mixed naïve bayes underestimated the fraction of positives in lower ranges of predicted probabilities (< 60%) in spontaneous vaginal birth, but overestimated it in the higher ranges of predicted probabilities (> 60%) (Fig. 2, Calibration Curve). The models for vaginal operative delivery underestimated the fraction of positives throughout the range of predicted probabilities (Fig. 2).

A perfect classifier would predict probabilities near 0% (e.g. no cesarean section) and near 100% (e.g. cesarean section is very likely)^33^. None of the models predicted the outcomes with high certainty, with probabilities either below 50% (cesarean section, vaginal operative delivery), or distributed around 50% (spontaneous vaginal birth) (Fig. 2).

At a 50% threshold, the mixed naïve bayes (study group 1: 15%) and random forest classifier (SG1: 10%) showed the highest sensitivity in predicting cesarean section with an increasing trend along the study groups (Table 2, Table S4 supplementary material). Yet, sensitivity stayed below 20%. In contrast, for spontaneous vaginal birth sensitivity reached 91% using a neural network and 90% with logistic regression with a decreasing trend along the study groups (Table 2, Table S4 supplementary material). However, specificity was under 34% and the PPV only reached a maximum of 67%.

Balanced accuracy ranged between 52% and 57% for spontaneous vaginal birth and cesarean section with all classifiers (Table 2, Table S4 supplementary material). Vaginal operative delivery could not be predicted with any of the algorithms (balanced accuracy = 50%). The support vector machine classifier model also failed to predict any of the outcomes.

The performance metrics did not differ relevantly in the study groups (Table S4, Fig. S7-S9 supplementary material).

The neural network, as being one of the best performing algorithms in the binary outcome prediction, was chosen to predict the multiclass outcome. The overall performance regarding auROC and auPR was comparable to the results obtained from the separate binary classification tasks across all study groups (Fig. S10-S13). While sensitivity, specificity, and positive predictive value differed slightly from those based on the fixed 50% threshold used in the binary classification (Table S5), this is expected in multiclass classification, where a direct threshold is not applied. Instead, the predicted class per observation is determined by selecting the class with the highest predicted probability.

## Discussion

To our knowledge, this is the first study predicting mode of delivery in a population of prolonged pregnancies using machine learning methods. Routinely collected prospective data from the population-based Swedish MBR were processed to create different study groups for increasing gestational ages. This reflects the clinical situation when a decision should be made on induction of labour or expectant management in healthy prolonged pregnancies (≥ 41 GW). In this population, different models predicted spontaneous vaginal birth, cesarean section and vaginal operative delivery, respectively. The rates for these outcomes were imbalanced. Variables registered until the day of decision-making were used as predictive features. Even the decision itself (IOL or EM) could be considered in the models by including the corresponding binary variable. However, the predictive power of the features derived was low and the chosen maternal outcomes could not be predicted with high certainty.

A major strength of the study is the use of a large, population-based cohort with a nationwide coverage and a prospective data collection^22^. In line with clinical management and current research from RCTs^9,27,28^ and observational studies (e.g^7,8^.) both women who were induced at a certain threshold (IOL) and women who delivered after the respective threshold (EM) were included in the respective study groups.

However, some misclassification error may have occurred because the actual date of the initiation of IOL is not registered in the MBR and the day of admission to the hospital as the best possible proxy was used instead. Nevertheless, women who were admitted to the hospital and induced more than one day later than the admission could be misclassified in the respective IOL group. To account for this potential misclassification, women with an antenatal stay beyond five days were excluded. This was underpinned by the fact, that 98% of the women in our study population delivered between day 0 and day 5 after the day of admission (data not shown).

Further misclassification could have been possible in the IOL group because the indication for induction is not registered in the MBR. To simulate an RCT and reflect clinical management, women who were grouped into IOL should have been induced due to the gestational age, and not because of other medical reasons. This is why women with e.g. a registered diagnosis of preeclampsia in the IOL group were excluded although this could also be a case of a postpartum preeclampsia and not the reason for the induction.

The lack of indication for induction could have led to a bias towards an IOL group with more pathologies than the EM group. During the study period Swedish Guidelines recommended EM until 42 GW in the absence of any risk factors^34^. Nevertheless, rates for induction of labour in week 41 increased from 7,7% in 1999 to 43,6% in 2022 with a doubling of the rate after the Swedish RCT on labour induction at 41 weeks (SWEPIS trial^28^ in 2020^35^. It cannot be completely ruled out that some of the pregnancies classified as IOL at least in study group 1 and 2 before 2020 were induced because of an underlying complication (“confounding by indication”). However, as the rate of IOL was < 5% in study group 1–3 (Table 1) it can be assumed that these cases happened not very frequently.

One of the major technical issues in prediction studies on medical outcomes are the low outcome rates which cause imbalanced data sets. Class imbalance occurs if the number of observations with the outcome is unequal to the number of observations without the outcome^36^. In this case, the algorithms tend to be biased towards the majority class.

The imbalanced data sets explain in part the pattern of the performance metrics in the present study, including calibration curves and the distribution of the predicted probabilities.

The values for the auROCs reached a maximum of 70%. Hence, the algorithms could not distinguish well between the positive and the negative class. This applies to all models and study groups (Table 2, Table S4 supplementary material).

Precision-recall curves can be more robust compared to ROC curves in imbalanced data sets as they evaluate the fraction of true positives among the positive predictions^37^. In spontaneous vaginal birth, the auPR more than doubled compared to the auPR of cesarean section. However, it has to be taken into consideration that the rate of spontaneous vaginal birth was approximately 2–3 times higher than the rate for cesarean section (Table 1).

The uncertainty of the prediction models in the present study is supported by the distribution of the predicted probabilities (Fig. 2, histograms). Depending on the chosen algorithm, a perfectly calibrated classifier has two peaks in the distribution for predicted probabilities, one close to 0% and one close to 100%^33^. For spontaneous vaginal birth the predicted probabilities were grouped around 50%, with a high uncertainty in the prediction of the classes. For cesarean section and vaginal operative delivery, where the outcome rates were similar (between 17% and 27%) (Table 1.), the majority of the probabilities group below 50%. Hence, the algorithms tend to predict the negative class with higher probabilities. This is also reflected by the numbers in the corresponding confusion matrices, where mainly the majority class was predicted (data not shown).

We did not apply methods for correction of the class imbalance in this study. Current research implies, that it should not be recommended to enhance the minority class in prediction models with observational data as the performance might even become worse^38^.

On the other hand, it can be assumed that the included features provide some predictive information beyond only mapping imbalanced data sets. The rates for vaginal operative delivery and cesarean section did not differ substantially but the auROC (> 60%) and auPR (35%) was higher while predicting cesarean section (Table 2; Fig. 2, Table S4 supplementary material). Future research should investigate the contribution of each feature to the prediction in order to quantify this assumption.

The low performance metrics observed are in line with current research. A recently published study on adverse perinatal outcomes in nulliparous women based on data from a prospective U.S.-cohort study had auROCs with a maximum of 0.673 (95% confidence interval: 0.651–0.694)^39^. The authors describe the difficulty of predicting adverse perinatal outcomes based on the results from previously published studies with similar auROCs e.g^14,40–42^.,.

However, in a study by Malacova et al.^11^ the auROC of ensemble classifiers outperformed other classifiers and reached 84% in predicting stillbirth (outcome rate < 1%). The rates for the PPV was not higher than 5% and much lower than in the present study. Though, these values cannot directly be compared to the values in the present study as the authors did not apply a threshold of 50% but an FPR of 5% and 10%. The authors did not provide a precision-recall curve and corresponding auPR.

Artzi and colleagues^12^ also described an auROC with a size of 85% from an XGBoost model in a study from 2020. The authors used data from medical records of early pregnancy to predict gestational diabetes (outcome rate 4%) at an earlier stage than the usual 28 GW. In contrast, the auPR did not exceed 32% which was comparable to the auPR of predicting cesarean section in the current study.

As the present study is of exploratory design aiming for high performing prediction models, we did not apply methods of feature ranking or causal inference.

Methods of feature ranking like impurity-based feature importance, permutation importance or Shapley values (SHapley Additive exPlanations) have the potential to generate insights into the algorithms’ decisions by calculating values for the feature importance.

While impurity-based feature importance of random forests can be misleading for high cardinality features^43^ which are also represented in the data set (e.g. age, height, BMI) permutation importance depends on the chosen classification metric and could be inaccurate in imbalanced data sets^44^ like in the present study. Moreover, the feature ranking can also differ depending on the performance of the model, which means that one feature can be important in one model, but unimportant in another model^44^. Given the high clinical impact of the decision on IOL or EM a feature ranking should be based on a high performing model, especially for the implementation in a clinical setting. However, to understand why the model performance in obstetric research is often very low^39^ and which features are contributing to the prediction, different methods of feature rankings (e.g. permutation importance and Shapley values) should be applied and compared.

The effect of IOL in late pregnancies on perinatal health is still under discussion and there is a strong need to isolate the effect of this intervention in term pregnancies. G-computation is a method for estimating causal effects from observational data by modelling the expected outcome under different exposure scenarios^45^. It recently has been combined with machine learning methods to quantify the effect of a treatment^46^. This could also be a promising approach to estimate the effect of IOL in late pregnancies on perinatal health, but requires high performing q-models^47^.

There is evidence that the performance of prediction studies for adverse perinatal outcomes can be improved by adding more relevant features to the models^11,39^. In the present study, data from the MBR was used. The available and eligible variables (known on the day of decision-making) include information on diseases, maternal anthropometrics before pregnancy and few socioeconomic factors with assumable limited effect on the outcomes (Table 1). Including more granular data from other registers could enhance predictive power of the models.

## Conclusion

We predicted mode of delivery in nulliparous women with a prolonged pregnancy considering the clinically relevant decision-making process on IOL or EM. Predictive power of the included features was low and all models failed to predict the outcomes with high certainty. Including more granular clinical data could potentially address the problem of the lack of information for this prediction.

## Supplementary Information

Below is the link to the electronic supplementary material.

Supplementary Material 1

## Data Availability

The register data analysed during the current study are not publicly available due to the European General Data Protection Regulation 2016/6792 and the Swedish Data Protection Act (2018:218) containing supplementary provisions to the EU General Data Protection Regulation but are available from the corresponding author on reasonable request and with permission of the National Board of Health and Welfare (Socialstyrelsen).

## Code availability

The code for data-preprocessing steps only applies to the used, sensitive data and is therefore not published. The code used for the machine learning algorithms is derived from open-source Python packages (e.g. scikit-learn, numpy, pandas, tensorflow, mixed naïve bayes) with the respective documentation and example code.

## References

[1] Muglu, J. et al. Risks of stillbirth and neonatal death with advancing gestation at term: A systematic review and meta-analysis of cohort studies of 15 million pregnancies. *PLoS Med.* **16** (7), e1002838 (2019). 31265456 10.1371/journal.pmed.1002838 PMC6605635

[2] WHO Guidelines. Approved by the guidelines review committee, in WHO Recommendations on Induction of Labour at or Beyond Term. World Health Organization: Geneva. (2022).

[3] Middleton, P. et al. Induction of labour at or beyond 37 weeks’ gestation. *Cochrane Database Syst. Rev.* **7** (7), pCd004945 (2020). 10.1002/14651858.CD004945.pub5 PMC7389871 32666584

[4] Jeer, B. et al. Perinatal and maternal outcomes according to timing of induction of labour: A systematic review and meta-analysis. *Eur. J. Obstet. Gynecol. Reproductive Biology*. **288**, 175–182 (2023). 10.1016/j.ejogrb.2023.07.021 37549509

[5] Alkmark, M. et al. Induction of labour at 41 weeks or expectant management until 42 weeks: A systematic review and an individual participant data meta-analysis of randomised trials. *PLoS Med.* **17** (12), e1003436 (2020). 33290410 10.1371/journal.pmed.1003436 PMC7723286

[6] Bruinsma, A. et al. Elective induction of labour and expectant management in late-term pregnancy: A prospective cohort study alongside the INDEX randomised controlled trial. *Eur. J. Obstet. Gynecol. Reprod. Biol. X*. **16**, 100165 (2022). 36262791 10.1016/j.eurox.2022.100165 PMC9574420

[7] Pyykönen, A. et al. Propensity score method for analyzing the effect of labor induction in prolonged pregnancy. *Acta Obstet. Gynecol. Scand.* **97** (4), 445–453 (2018). 28832917 10.1111/aogs.13214

[8] Ravelli, A. C. J. et al. Does induction of labor at 41 weeks (early, mid or late) improve birth outcomes in low-risk pregnancy? A nationwide propensity score-matched study. *Acta Obstet. Gynecol. Scand.* **102** (5), 612–625 (2023). 36915238 10.1111/aogs.14536 PMC10072249

[9] Keulen, J. K. et al. Induction of labour at 41 weeks versus expectant management until 42 weeks (INDEX): multicentre, randomised non-inferiority trial. *Bmj* **364**, l344 (2019). 30786997 10.1136/bmj.l344 PMC6598648

[10] Jelovsek, J. E. et al. Predicting risk of pelvic floor disorders 12 and 20 years after delivery. *Am. J. Obstet. Gynecol.* **218** (2), 222e1–222e19 (2018). 10.1016/j.ajog.2017.10.014 29056536

[11] Malacova, E. et al. Stillbirth risk prediction using machine learning for a large cohort of births from Western australia, 1980–2015. *Sci. Rep.* **10** (1), 5354 (2020). 32210300 10.1038/s41598-020-62210-9 PMC7093523

[12] Artzi, N. S. et al. Prediction of gestational diabetes based on nationwide electronic health records. *Nat. Med.* **26** (1), 71–76 (2020). 31932807 10.1038/s41591-019-0724-8

[13] The fetal medicine foundation. ; (2024). Available from: https://www.fetalmedicine.org/research/assess/preeclampsia/first-trimester

[14] Trudell, A. S. et al. A stillbirth calculator: development and internal validation of a clinical prediction model to quantify stillbirth risk. *PLoS One*. **12** (3), e0173461 (2017). 28267756 10.1371/journal.pone.0173461 PMC5340400

[15] Gimovsky, A. C. et al. Pushing the bounds of second stage in term Nulliparas with a predictive model. *Am. J. Obstet. Gynecol. MFM*. **1** (3), 100028 (2019). 33345792 10.1016/j.ajogmf.2019.07.001

[16] Tsur, A. et al. *Development and Validation of a machine-learning Model for Prediction of Shoulder Dystocia*56 (Ultrasound in Obstetrics & Gynecology, 2020). 10.1002/uog.21878 31587401

[17] *TRIPOD + AI Expanded Checklist (Explanation & Elaboration Light)*. Available from: https://www.tripod-statement.org/wp-content/uploads/2024/04/TRIPODAI-Supplement.pdf

[18] Collins, G. S. et al. TRIPOD + AI statement: updated guidance for reporting clinical prediction models that use regression or machine learning methods. *BMJ* **385**, e078378 (2024). 38626948 10.1136/bmj-2023-078378 PMC11019967

[19] Cnattingius, S. et al. The Swedish medical birth register during five decades: Documentation of the content and quality of the register. *Eur. J. Epidemiol.* **38** (1), 109–120 (2023). 36595114 10.1007/s10654-022-00947-5 PMC9867659

[20] Ludvigsson, J. F. et al. The Swedish personal identity number: possibilities and pitfalls in healthcare and medical research. *Eur. J. Epidemiol.* **24** (11), 659–667 (2009). 19504049 10.1007/s10654-009-9350-y PMC2773709

[21] Brooke, H. L. et al. The Swedish cause of death register. *Eur. J. Epidemiol.* **32** (9), 765–773 (2017). 28983736 10.1007/s10654-017-0316-1 PMC5662659

[22] Ludvigsson, J. F. et al. Registers of the Swedish total population and their use in medical research. *Eur. J. Epidemiol.* **31** (2), 125–136 (2016). 26769609 10.1007/s10654-016-0117-y

[23] Wettermark, B. et al. The new Swedish prescribed drug Register–opportunities for pharmacoepidemiological research and experience from the first six months. *Pharmacoepidemiol Drug Saf.* **16** (7), 726–735 (2007). 16897791 10.1002/pds.1294

[24] Cnattingius, S. et al. Maternal obesity and risk of preterm delivery. *Jama* **309** (22), 2362–2370 (2013). 23757084 10.1001/jama.2013.6295

[25] Johansson, K. et al. Risk of pre-eclampsia after gastric bypass: a matched cohort study. *Bjog* **129** (3), 461–471 (2022). 34449956 10.1111/1471-0528.16871

[26] Stephansson, O. et al. Delivery outcomes in term births after bariatric surgery: Population-based matched cohort study. *PLoS Med.* **15** (9), e1002656 (2018). 30256796 10.1371/journal.pmed.1002656 PMC6157842

[27] Grobman, W. A. et al. Labor induction versus expectant management in Low-Risk nulliparous women. *N. Engl. J. Med.* **379** (6), 513–523 (2018). 30089070 10.1056/NEJMoa1800566 PMC6186292

[28] Wennerholm, U. B. et al. Induction of labour at 41 weeks versus expectant management and induction of labour at 42 weeks (SWEdish Post-term induction study, SWEPIS): multicentre, open label, randomised, superiority trial. *Bmj* **367**, l6131 (2019). 31748223 10.1136/bmj.l6131 PMC6939660

[29] Socialstyrelsen *Framställning och kvalitet - medicinska födelseregistret (by the Swedish Board of National Health and Welfare)*. ; (2021). Available from: https://www.socialstyrelsen.se/globalassets/sharepoint-dokument/artikelkatalog/statistik/2021-9-7547.pdf

[30] Bhaskaran, K. & Smeeth, L. What is the difference between missing completely at random and missing at random? *Int. J. Epidemiol.* **43** (4), 1336–1339 (2014). 24706730 10.1093/ije/dyu080 PMC4121561

[31] George, L. et al. Self-reported nicotine exposure and plasma levels of cotinine in early and late pregnancy. *Acta Obstet. Gynecol. Scand.* **85** (11), 1331–1337 (2006). 17091413 10.1080/00016340600935433

[32] Mixed Naive *Bayes*. Available from: https://pypi.org/project/mixed-naive-bayes/

[33] Niculescu-Mizil, A. & Caruana, R. *Predicting good probabilities with supervised learning*, in *Proceedings of the 22nd international conference on Machine learning*. Association for Computing Machinery: Bonn, Germany. pp. 625–632. (2005).

[34] Svensk Förening för Obstetrik & Gynekologi (SFOG). Available from: https://www.sfog.se/start/kunskapsstoed/obstetrik/foerlossning/

[35] The National Board of Health and Welfare. *Official Statistics of Sweden. Statistics – Health and Medical Care. Pregnancies, Deliveries and Newborn Infants. The Swedish Medical Birth Register 1973–2022.* Novemebr 22, 2024]; Available from: https://www.socialstyrelsen.se/statistik-och-data/statistik/alla-statistikamnen/graviditeter-forlossningar-och-nyfodda/

[36] Megahed, F. M. et al. The class imbalance problem. *Nat. Methods*. **18** (11), 1270–1272 (2021). 34654918 10.1038/s41592-021-01302-4

[37] Saito, T. & Rehmsmeier, M. The Precision-Recall plot is more informative than the ROC plot when evaluating binary classifiers on imbalanced datasets. *PLOS ONE*. **10** (3), e0118432 (2015). 25738806 10.1371/journal.pone.0118432 PMC4349800

[38] van den Goorbergh, R. et al. The harm of class imbalance corrections for risk prediction models: illustration and simulation using logistic regression. *J. Am. Med. Inf. Assoc.* **29** (9), 1525–1534 (2022). 10.1093/jamia/ocac093 PMC9382395 35686364

[39] Lee, S. J. et al. Interpretable machine learning to predict adverse perinatal outcomes: examining marginal predictive value of risk factors during pregnancy. *Am. J. Obstet. Gynecol. MFM*. **5** (10), 101096 (2023). 37454734 10.1016/j.ajogmf.2023.101096

[40] Lee, K. S. & Ahn, K. H. Artificial neural network analysis of spontaneous preterm labor and birth and its major determinants. *J. Korean Med. Sci.* **34** (16), e128 (2019). 31020816 10.3346/jkms.2019.34.e128 PMC6484180

[41] Park, S. et al. Predicting preterm birth through vaginal microbiota, cervical length, and WBC using a machine learning model. *Front. Microbiol.* **13**, 912853 (2022). 35983325 10.3389/fmicb.2022.912853 PMC9378785

[42] Yerlikaya, G. et al. Prediction of stillbirth from maternal demographic and pregnancy characteristics. *Ultrasound Obstet. Gynecol.* **48** (5), 607–612 (2016). 27561693 10.1002/uog.17290

[43] scikit-learn developers. Feature importances with a forest of trees - scikit-learn 1.70 documentation. [cited 2025 12 June]; (2024). Available from: https://scikit-learn.org/stable/auto_examples/ensemble/plot_forest_importances.html

[44] scikit-learn developers. 5.2. Permutation feature importance - scikit-learn 1.7.0 documentation. [cited 2025 June 12]; (2024). Available from: https://scikit-learn.org/stable/modules/permutation_importance.html

[45] Robins, J. A new approach to causal inference in mortality studies with a sustained exposure period—application to control of the healthy worker survivor effect. *Math. Modelling*. **7** (9–12), 1393–1512 (1986).

[46] Le Borgne, F. et al. G-computation and machine learning for estimating the causal effects of binary exposure statuses on binary outcomes. *Sci. Rep.* **11** (1), 1435 (2021). 33446866 10.1038/s41598-021-81110-0 PMC7809122

[47] Snowden, J. M., Rose, S. & Mortimer, K. M. Implementation of G-computation on a simulated data set: demonstration of a causal inference technique. *Am. J. Epidemiol.* **173** (7), 731–738 (2011). 21415029 10.1093/aje/kwq472 PMC3105284

