# Supplementary material for: Machine learning prediction models for mode of delivery in prolonged pregnancies in Sweden
Source: Sci Rep. 2025 Sep 12;15:32487. doi: 10.1038/s41598-025-19198-x (PMC12432169; doi:10.1038/s41598-025-19198-x)
Supplement: Supplementary file 1 — Supplementary Material 1 [file 41598_2025_19198_MOESM1_ESM.docx]

**Supplementary material**

Legend:

**Figure S1:** Flow chart of the initial study population.

**Figure S2:** Flow Chart of Study group 1

**Figure S3**. Flow Chart Study group 2

**Figure S4:** Flow Chart Study group 3

**Figure S5:** Flow Chart Study group 4

**Figure S6:** Construction of the study groups 2-4 and the resulting binary variable “induction of labour”.

**Figure S7:** Performance metrics (ROC Curves, PR Curves, Calibration Curves) of the models in study group 2 (SG2).

**Figure S8:** Performance metrics (ROC Curves, PR Curves, Calibration Curves) of the models in study group 3 (SG3).

**Figure S9:** Performance metrics (ROC Curves, PR Curves, Calibration Curves) of the models in study group 4

**Figure S10:** Performance metrics (ROC Curves, PR Curves, Calibration Curves) of the models in study group 1 (SG1) in the multiclass prediction with a neural network.

**Figure S11:** Performance metrics (ROC Curves, PR Curves, Calibration Curves) of the models in study group 2 (SG2) in the multiclass prediction with a neural network.

**Figure S12:** Performance metrics (ROC Curves, PR Curves, Calibration Curves) of the models in study group 3 (SG3) in the multiclass prediction with a neural network.

**Figure S13:** Performance metrics (ROC Curves, PR Curves, Calibration Curves) of the models in study group 4 (SG4) in the multiclass prediction with a neural network.

**Table S1:** Swedish version of the International Classification of Diseases (ICD)-10 Codes

**Table S2:** Prevalence of missing values in the variables for “smoking” and “snuff use” in the study population

**Table S3:** number of complete cases for each split of the feature set and the outcomes.

**Table S4:** Performance metrics of the different models in Study Group 1-4 (SG1-SG4) for all outcomes.

**Table S5:** Performance metrics of the different models in Study Group 1-4 (SG1-SG4) for all outcomes in the multiclass prediction with a neural network.

**Table S6:** Years of included data sources for the use as the MBR

**Table S7**: Variables of the MBR used in the analysis and the corresponding source.

**Table S8:** Variables calculated prior to the present study and the corresponding source or calculation

n = 2,971,004 pregnancies in the Medical Birth Register between

1992-2019

Exclusion:

n = 636,847 birth year < 1998

n = 2,334,157

Missing values*:

n = 36,348 (1.56 %) missing PIN of the mother

n = 2,837 (0.12 %) missing values for cesarean section

n = 1,454 (0.06 %) missing values length of gestation

n = 9,149 (0.39 %) missing values for date of admission

n = 20 (0.0 %) missing values for birth date of the infant

n = 3,494 (0.15 %) missing values for parity

n = 12,141 (0.52 %) missing values for onset of delivery

n = 2,279,031

Exclusion:

n = 25,119 (1,1 %) hospital admission until delivery < 0 or > 5 days (implausible)

n = 539,236

n = 2,253,912

Exclusion*:

n = 1,714,104 gestational age at day of admission < 287 days (41+0)

n = 60,283 multiple birth

n = 12,289 pre-existing diabetes

Exclusion:

n = 278,518 multiparous women

n = 260,718

nulliparous women

**Figure S1:** Flow chart of the initial study population. n = number of pregnancies. * no hierarchical exclusion.

n = 260,718

n = 201,446

n = 197,567

nulliparous women

Exclusion*:

n = 1,205 gestational diabetes

n = 1,559 breech

Exclusion when induced at 41+0/41+1*:

n = 990 preeclampsia

n = 95 bleeding antepartum (without a diagnose of placenta praevia)

n = 63 stillbirth before onset of labour

Exclusion:

n = 58,430 gestational age at admission 41+0/41+1 AND spontaneous birth

n = 842 gestational age at admission 41+0/41+1 AND elective cesarean

**Figure S2:** Flow Chart of Study group 1 (Induction of labour 41+0/1 or Expectant management > 41+1). * * no hierarchical exclusion.

n = 142,811

nulliparous women

Exclusion*:

n = 713 gestational diabetes

n = 1,077 breech

Exclusion when induced at 41+2/41+3*:

n = 788 preeclampsia

n = 75 bleeding antepartum (without a diagnose of placenta praevia)

n = 61 stillbirth before onset of labour

Exclusion:

n = 47,371 gestational age at admission 41+2/41+3 AND spontaneous birth

n = 715 gestational age at admission 41+2/41+3 AND elective cesarean

Exclusion:

n = 67,125 gestational age at day of admission < 41+2

n = 145,507

n = 193,593

n = 260,718

**Figure S3**. Flow Chart Study group 2 (IOL 41+2/3 or Expectant management > 41+3) * no hierarchical exclusion.

Exclusion*:

n = 498 gestational diabetes

n = 693 breech

Exclusion when induced at 41+4/41+5*:

n = 647 preeclampsia

n = 60 bleeding antepartum (without a diagnose of placenta praevia)

n = 43 stillbirth before onset of labour

Exclusion:

n = 36,907 gestational age at admission 41+4/41+5 AND spontaneous birth

n = 643 gestational age at admission 41+4/41+5 AND elective cesarean

Exclusion:

n = 121,526 gestational age at day of admission < 41+4

n = 101,642

n = 139,192

n = 260,718

n = 99,714

nulliparous women

**Figure S4:** Flow Chart Study group 3 (IOL 41+4/5 or Expectant management > 41+5). * no hierarchical exclusion.

Exclusion*

n = 323 gestational diabetes

n = 399 breech

Exclusion when induced at 41+6/42+0*:

n = 495 preeclampsia

n = 73 bleeding antepartum (without a diagnose of placenta praevia)

n = 42 stillbirth before onset of labour

n = 67,699

nulliparous women

n = 69,024

Exclusion:

n = 26,476 gestational age at admission 41+6/42+0 AND spontaneous birth

n = 661 gestational age at admission 41+6/42+0 AND elective cesarean

Exclusion:

n = 164,557 gestational age at day of admission < 41+6

n = 96,161

n = 260,718

**Figure S5:** Flow Chart Study group 4 (IOL 41+6/42+0 or Expectant management > 42+0). * no hierarchical exclusion.

a)


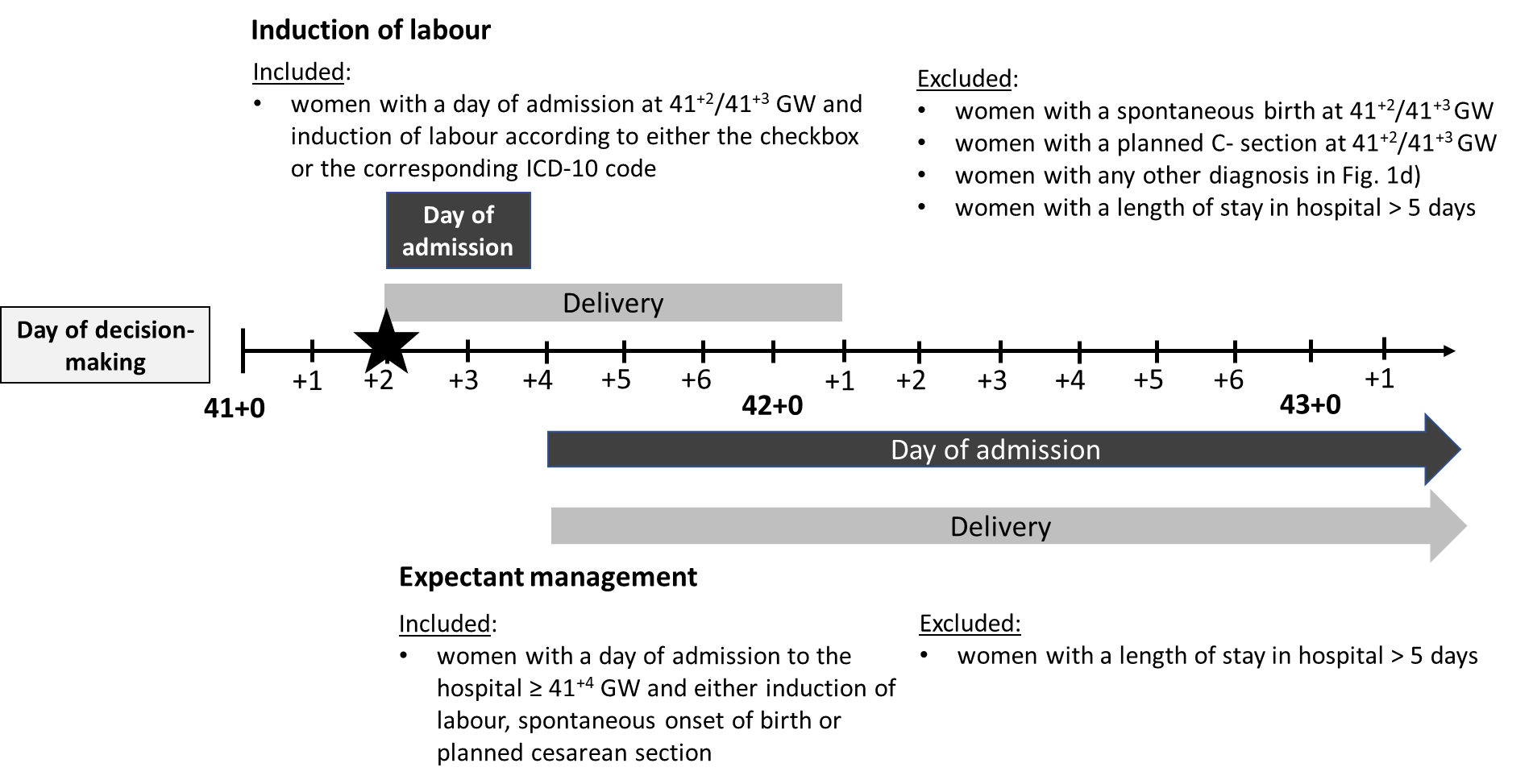


b)


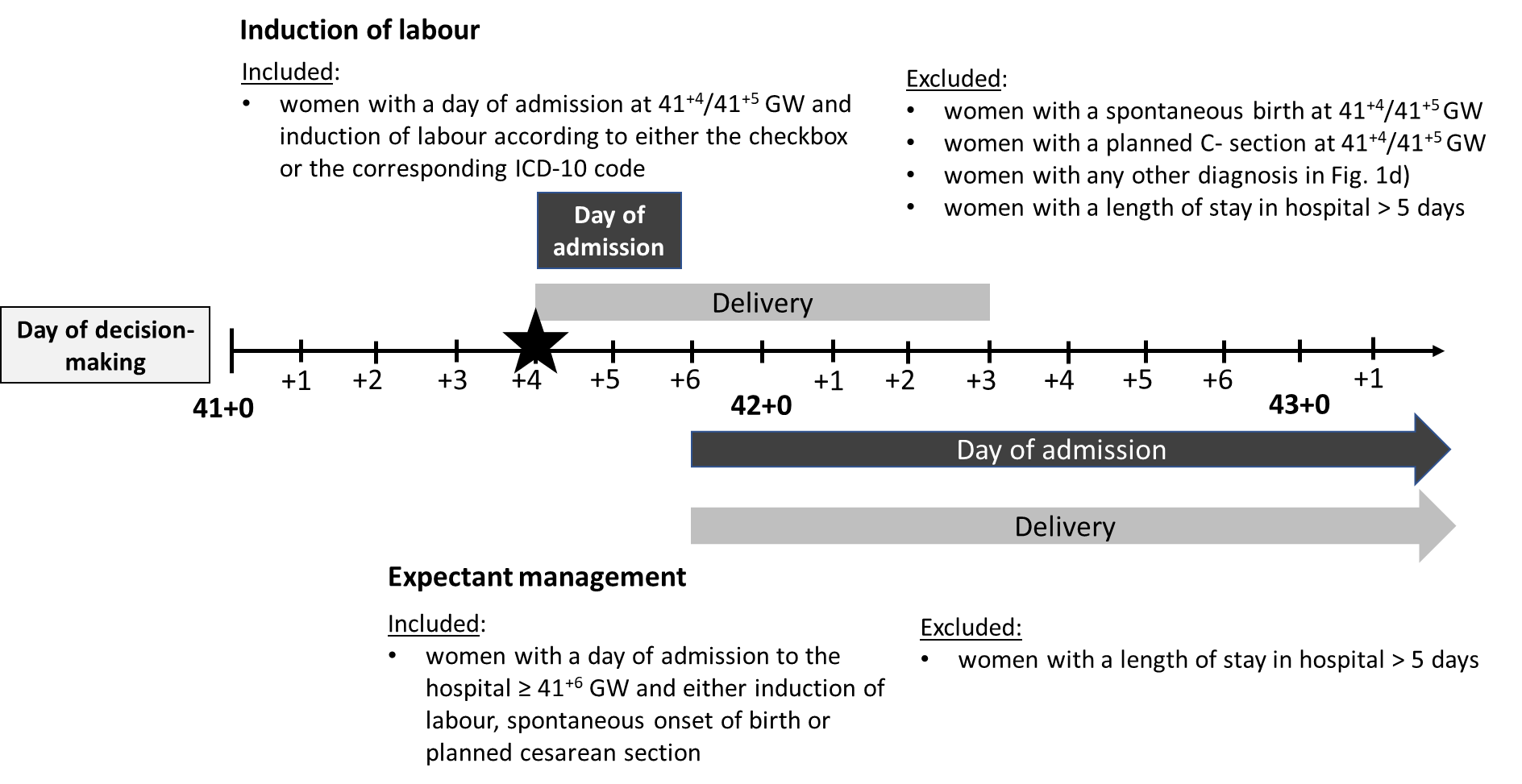


c)


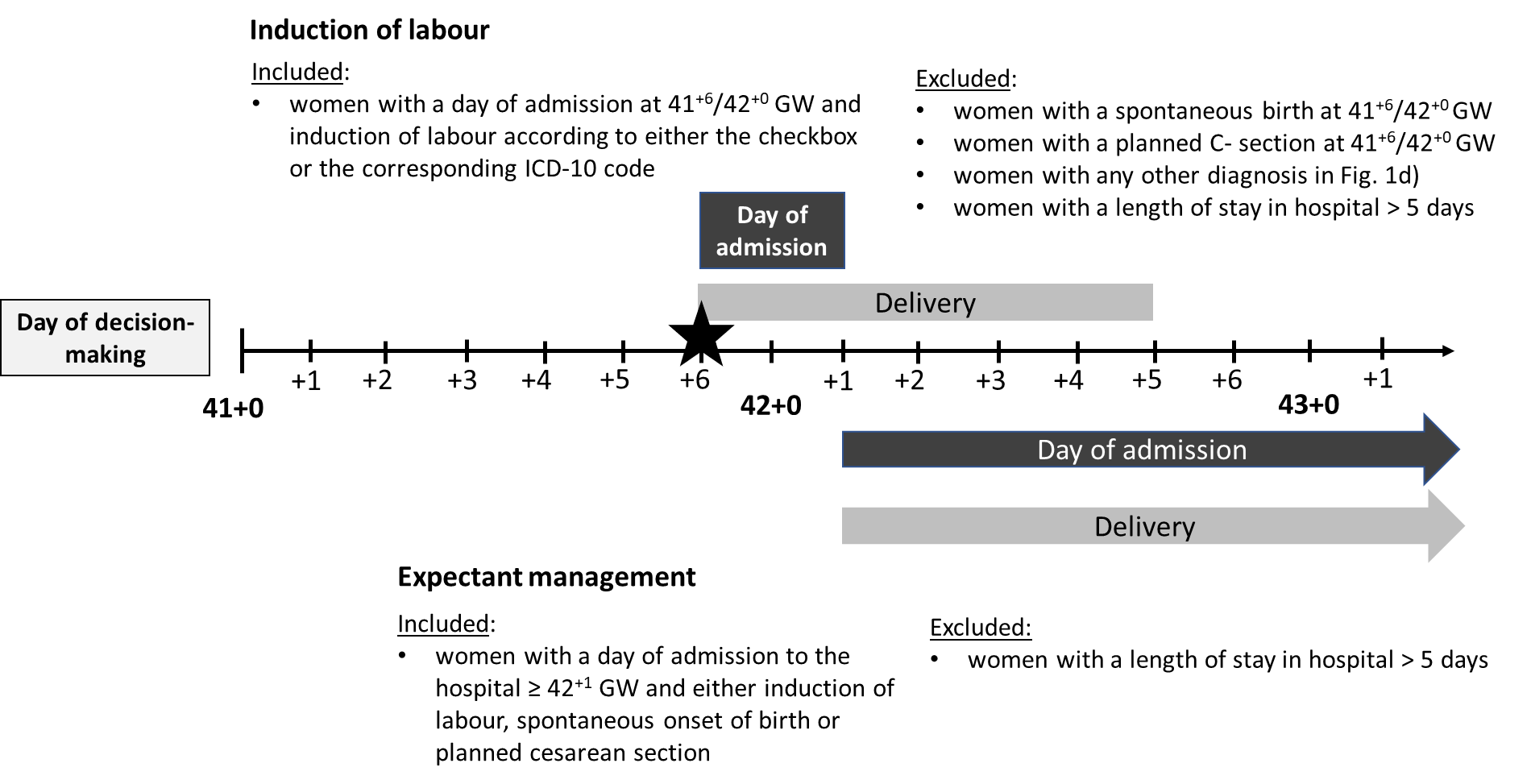


**Figure S6:** Construction of the study groups 2-4 and the resulting binary variable “induction of labour”. a) Study group 2 with a gestational age at day of admission and IOL of 41^+2/3^ GW, or EM with a gestational age at day of admission of ≥41^+4^ GW. **b)** Study group 3 with a gestational age at day of admission and IOL of 41^+4/5^ GW, or EM with a gestational age at day of admission of ≥41^+6^ GW. **c)** Study group 4 with a gestational age at day of admission and IOL of 41^+6/^42^+5^ GW, or EM with a gestational age at day of admission of ≥42^+1^ GW.

**Table S1:** Swedish version of the International Classification of Diseases (ICD)-10 Codes.

| **Diagnosis** | **corresponding ICD-10 code used in the study** |
| --- | --- |
| medical and mechanical induction of labour and delivery < 48 h or > 48h | O61.0A, O61.0B, O61.1A, O61.1B |
| PROM ≥ 37+0 GW and subsequent induction of labour (classified as spontaneous onset of labour) | O75.6B |
| PROM ≥ 37+0 GW spontaneous onset of birth; unspecified | O75.6A; O75.6X |
| elective cesarean section, cesarean section without medicial indication | O82.0, O82.8 |
| placental abruption | O45.0, O45.8, O45.9 |
| antenatal bleeding not classified elsewhere | O46.0, O46.8, O46.9 |
| placenta praevia (excluded) | O44.0, O44.1 |
| Diabetes Type 1 or 2 before pregnancy | O24.0, O24.0B, O24.0C, O24.0D, O24.0E, O24.0F, O24.0X, O24.1, O24.2, O24.3, E10-E14 |
| Gestational Diabetes | O24.4A, O24.B, O24.4X, O24.9 |
| Preeclampsia | O14, O15 |
| Preexisting hypertension | O10, O11, I10-I15 |
| Premature rupture of the membranes (22+0-36+6 GW) | 042 |

**Table S2:** Prevalence of missing values in the variables for “smoking” and “snuff use” in the study population (n = 260,718)

1. before replacement.

| **Variable** | **number of missing values (n)** | **%** |
| --- | --- | --- |
| Smoking before pregnancy | 25,073 | 9.6 |
| Smoking in early pregnancy | 12,629 | 4.8 |
| Smoking in late pregnancy | 48,188 | 18.5 |
| Snuff use before pregnancy | 22,291 | 8.5 |
| Snuff use in early pregnancy | 18,085 | 6.9 |
| Snuff use in late pregnancy | 130,745 | 50.1 |

b) after replacement.

| **Variable** | **number of missing values (n)** | **%** |
| --- | --- | --- |
| Smoking before pregnancy | 23,651 | 9.1 |
| Smoking in early pregnancy | 12,131 | 4.7 |
| Smoking in late pregnancy | 24,261 | 9.3 |
| Snuff use before pregnancy | 22,274 | 8.5 |
| Snuff use in early pregnancy | 17,519 | 6.7 |
| Snuff use in late pregnancy | 25,889 | 9.9 |

Values were replaced by using present values in other time points of smoking and snuff use and the assumption, that women do not start smoking or using snuff during pregnancy.

**Table S3:** number of complete cases for each split of the feature set and the outcomes.

|  | **SG1**  **(n = 178,932)** | **SG2**  **(n = 129,449)** | **SG3**  **(n = 90,448)** | **SG4**  **(n = 61,301)** |
| --- | --- | --- | --- | --- |
|  | **n** | **n** | **n** | **n** |
| X_train (70 %) | 125,252 | 90,614 | 63,313 | 42,910 |
| X_validate (10%) | 17,893 | 12,945 | 9,045 | 6,130 |
| X_test (20 %) | 35,787 | 25,890 | 18,090 | 12,261 |
| **a) cesarean section** | rate = 19.9 % | rate = 21.8 % | rate = 24.2 % | rate = 26.6 % |
| y_train (70 %) | 0 = 100,452  1 = 24,800 | 0 = 70,905  1 = 19,709 | 0 = 48,080  1 = 15,233 | 0 = 31,540  1 = 11,370 |
| y_validate (10%) | 0 = 14,350  1 = 3,543 | 0 = 10,129  1 = 2,816 | 0 = 6,869  1 = 2,176 | 0 = 4,506  1 = 1,624 |
| y_test (20 %) | 0 = 28,702  1 = 7,085 | 0 = 20,259  1 = 5,631 | 0 = 13,738  1 = 4,352 | 0 = 9,012  1 = 3,249 |
| **b) spontaneous birth** | rate = 63.2 % | rate = 60.9 % | rate = 58.4 % | rate = 55.9 % |
| y_train (70 %) | 0 = 45,953  1 = 79,299 | 0 = 35,292  1 = 55,322 | 0 = 26,257  1 = 37,056 | 0 = 18,907  1 = 24,003 |
| y_validate (10%) | 0 = 6,565  1 = 11,328 | 0 = 5,042  1 = 7,903 | 0 = 3,751  1 = 5,294 | 0 = 2,701  1 = 3,429 |
| y_test (20 %) | 0 = 13,129  1 = 22,658 | 0 = 10,083  1 = 15,807 | 0 = 7,503  1 = 10,587 | 0 = 5,402  1 = 6,859 |
| **c) vaginal operative delivery** | rate = 16.9 % | rate = 17.2 % | rate = 17.4 % | rate = 17.5 % |
| y_train (70 %) | 0 = 104,099  1 = 21,153 | 0 = 75,031  1= 15,583 | 0 = 52,288  1 = 11,025 | 0 = 35,373  1 = 7,537 |
| y_validate (10%) | 0 = 14,871  1 = 3,022 | 0 = 10,719  1 = 2,226 | 0 = 7,470  1 = 1,575 | 0 = 5,053  1= 1,077 |
| y_test (20 %) | 0 = 29,743  1 = 6,044 | 0 = 21,438  1 = 4,452 | 0 = 14,940  1 = 3,150 | 0 = 10,108  1 = 2,153 |


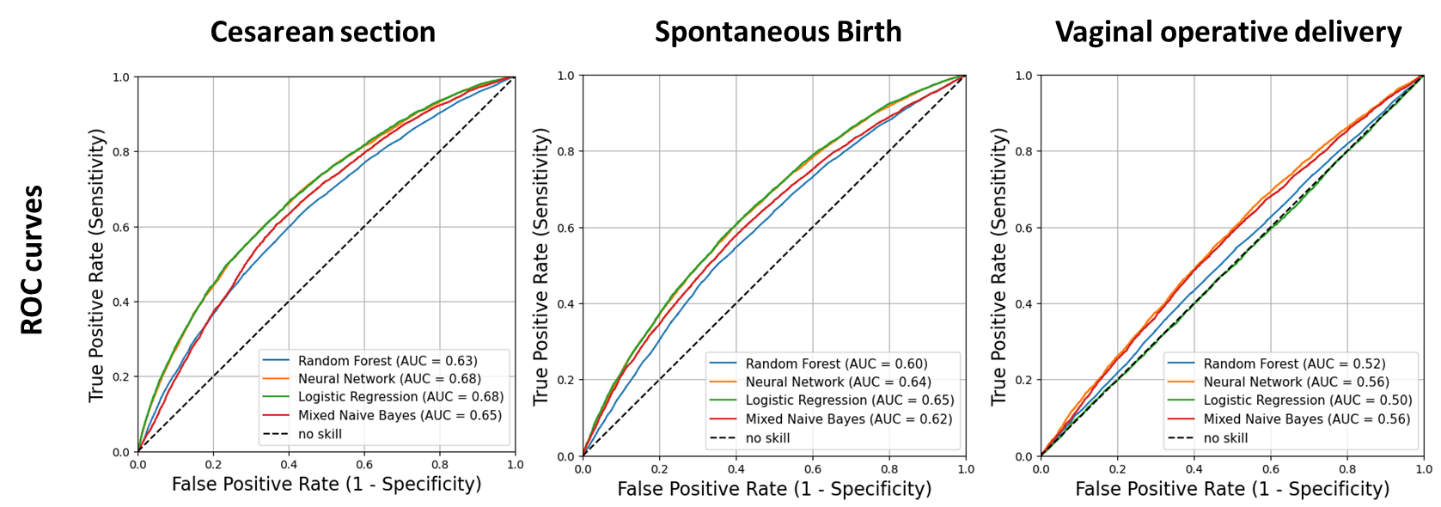


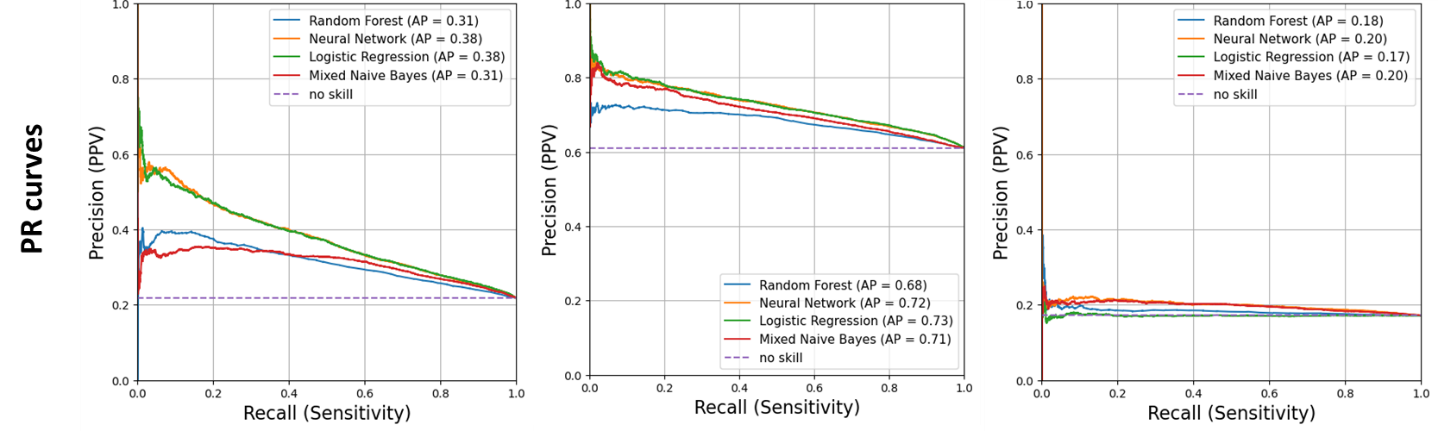


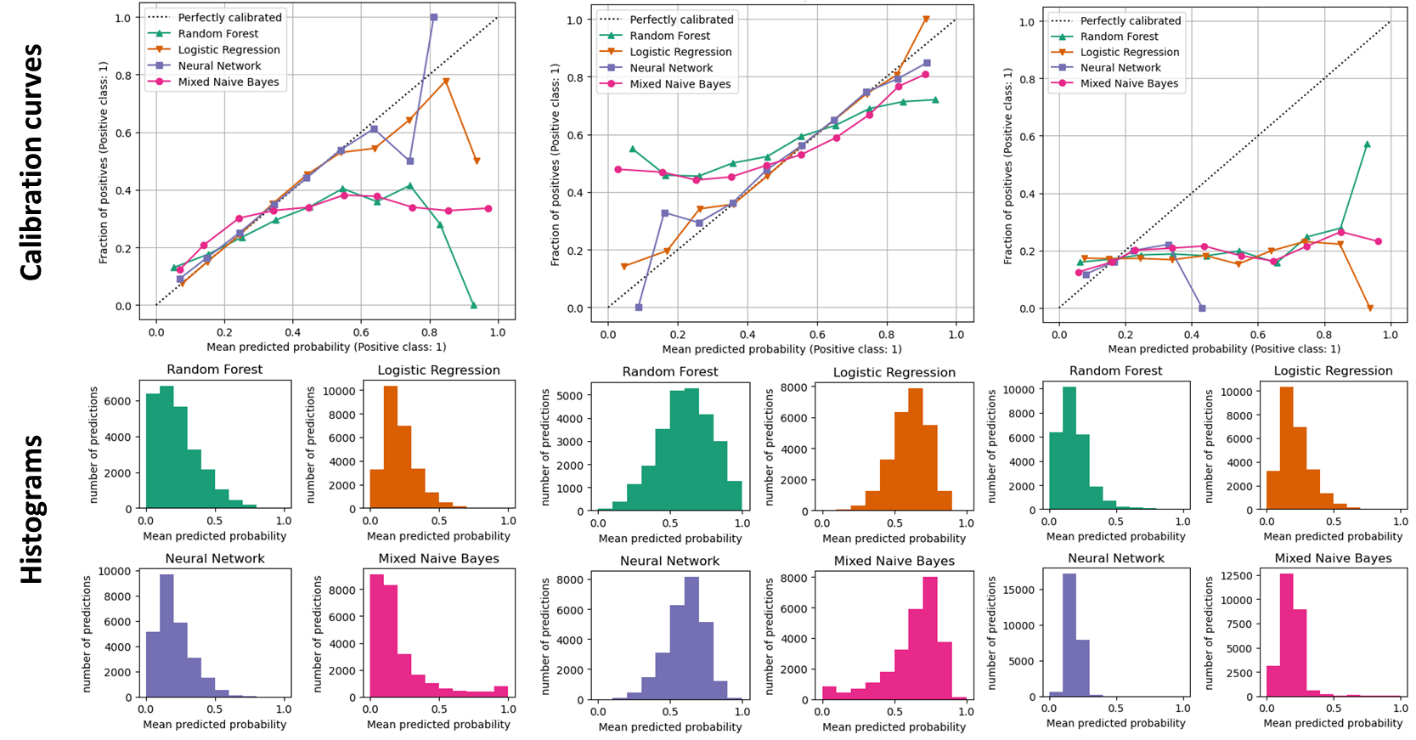


**Figure S7:** Performance metrics (ROC Curves, PR Curves, Calibration Curves) of the models in study group 2 (SG2). ROC curve: receiver operating characteristic curve; PR curve: Precision-Recall Curve; AUC: area under the ROC curve; AP: area under the precision recall curve.


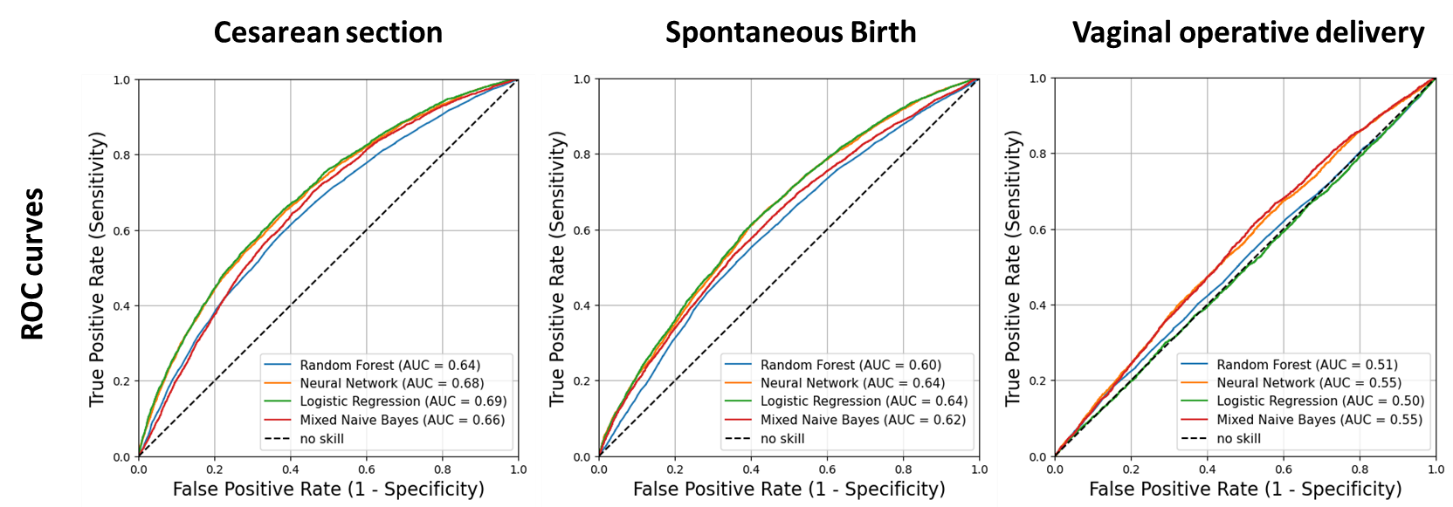


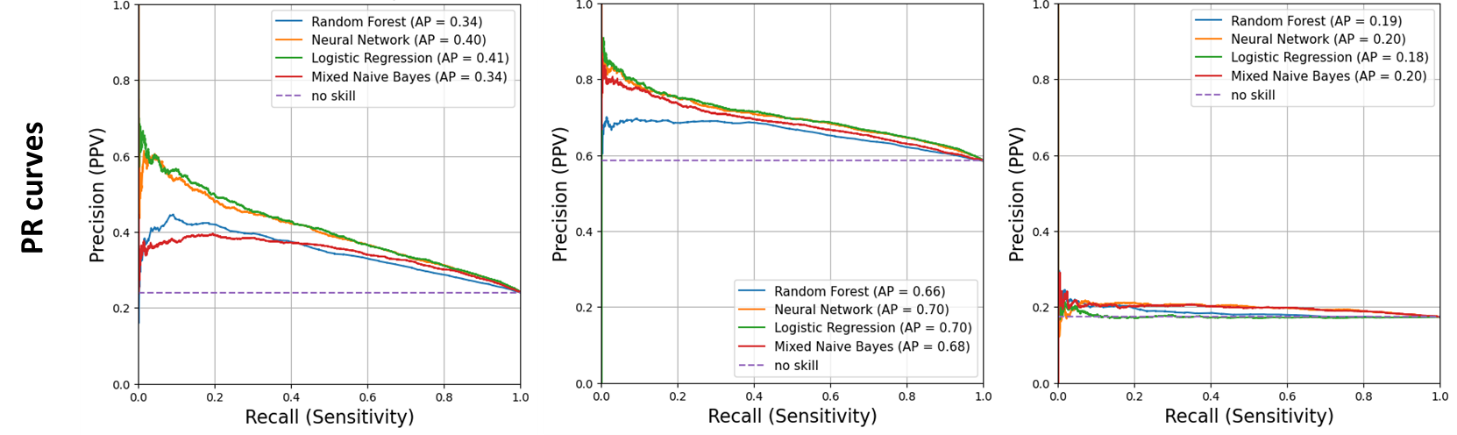


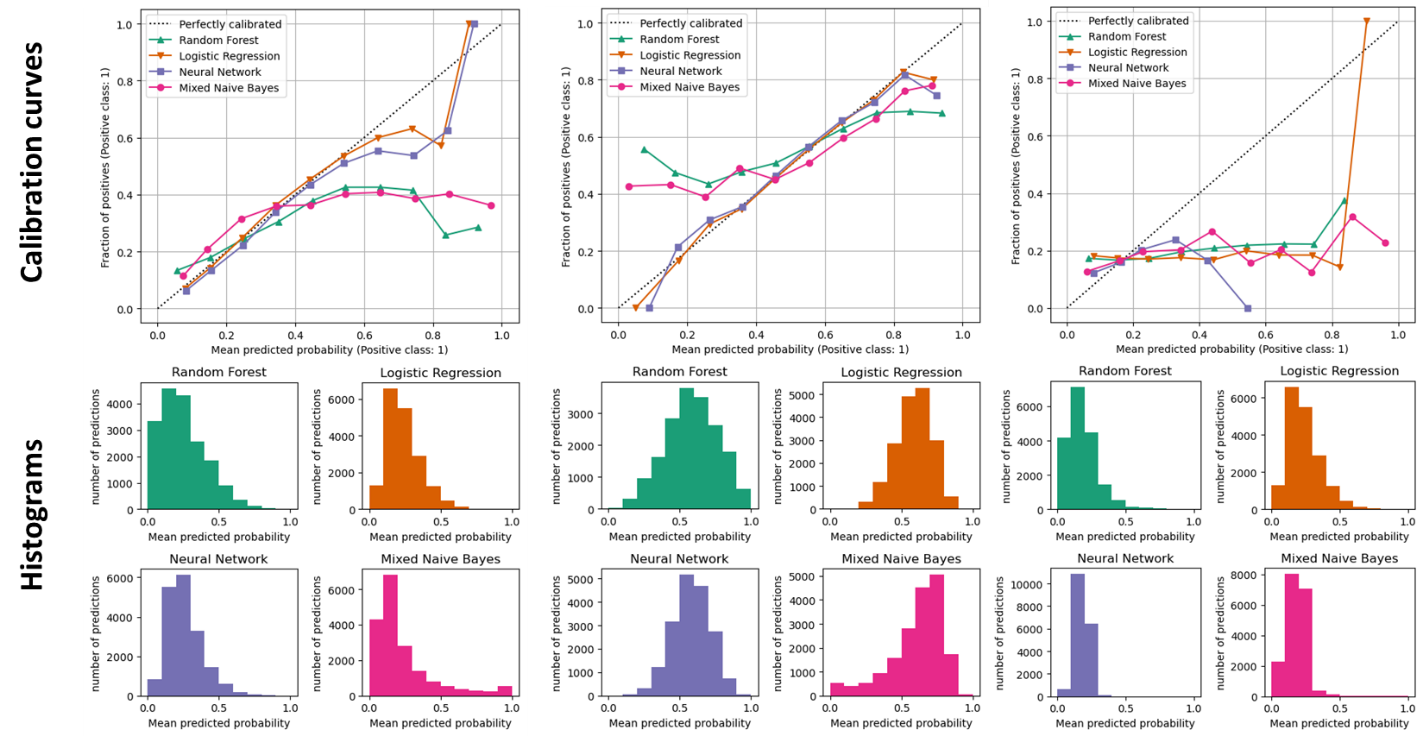


**Figure S8:** Performance metrics (ROC Curves, PR Curves, Calibration Curves) of the models in study group 3 (SG3). ROC curve: receiver operating characteristic curve; PR curve: Precision-Recall Curve; AUC: area under the ROC curve; AP: area under the precision recall curve.


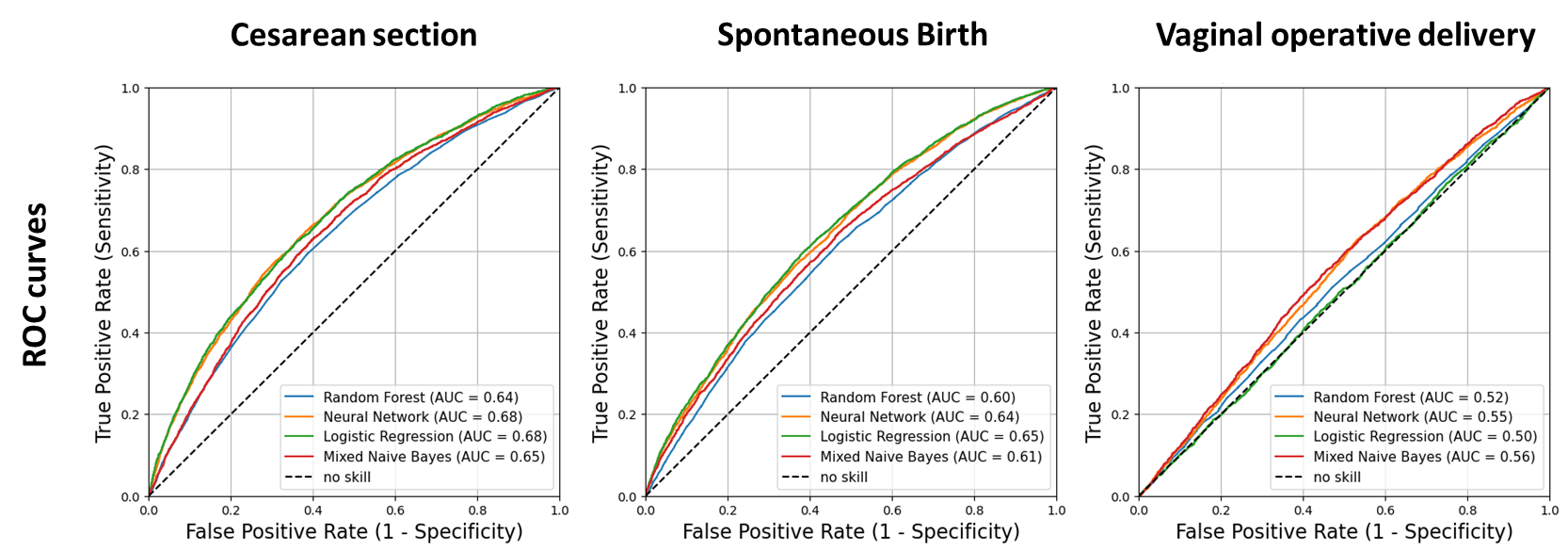


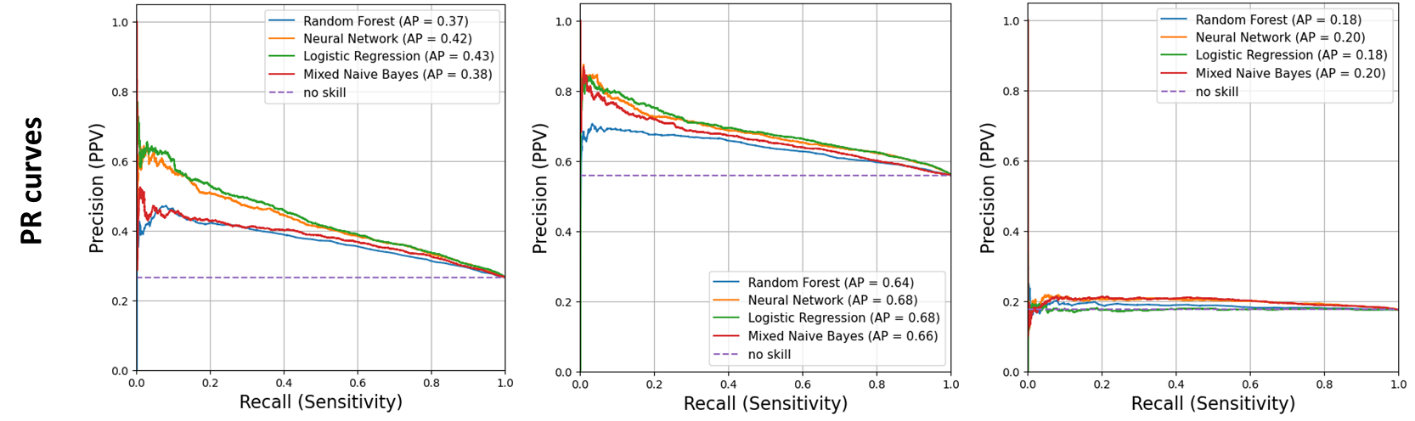


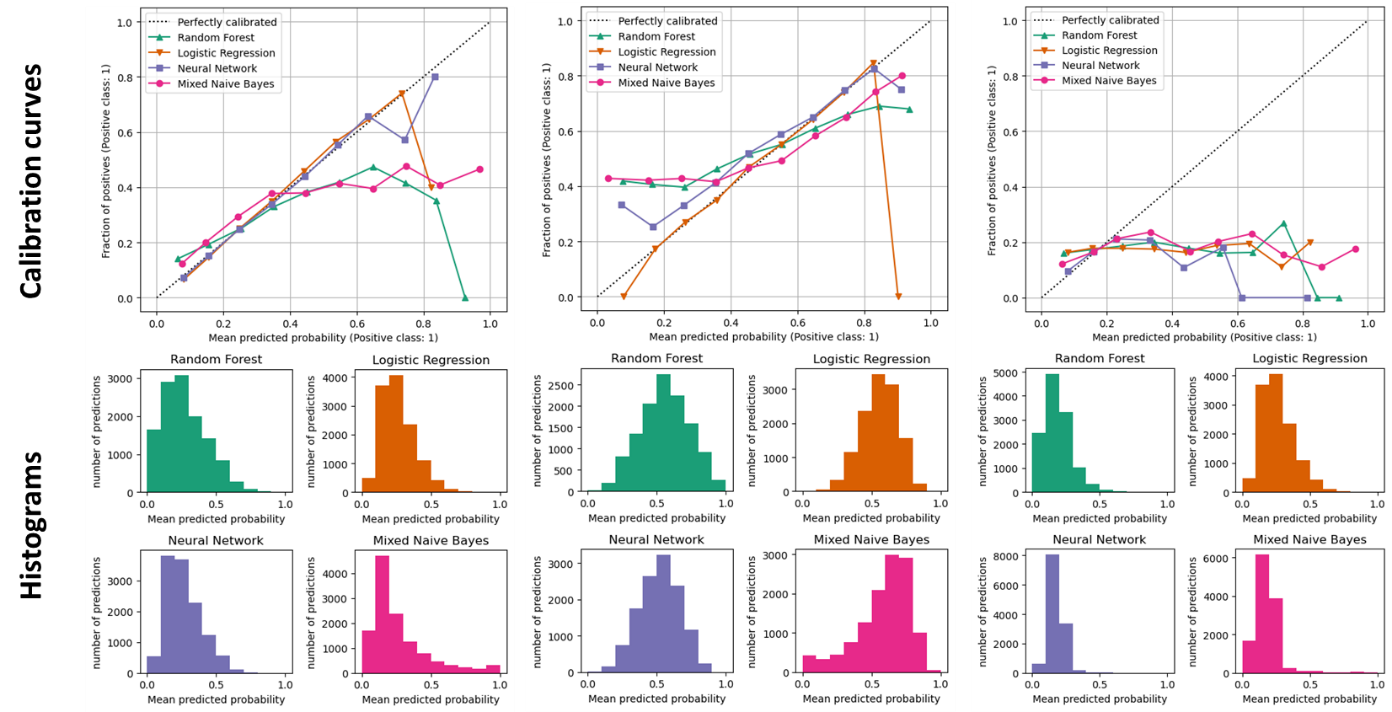


**Figure S9:** Performance metrics (ROC Curves, PR Curves, Calibration Curves) of the models in study group 4 (SG4). ROC curve: receiver operating characteristic curve; PR curve: Precision-Recall Curve; AUC: area under the ROC curve; AP: area under the precision recall curve.

**Table S4:** Performance metrics of the different models in Study Group 1-4 (SG1-SG4) for all outcomes.

| **a)** |  |  | **Study Group 1** | **Study Group 2** | **Study Group 3** | **Study Group 4** |
| --- | --- | --- | --- | --- | --- | --- |
| **CD** | **LR** | Sensitivity | 6 % | 6 % | 8 % | 10 % |
|  |  | Specificity | 99 % | 98 % | 98 % | 97 % |
|  |  | Precision | 53 % | 54 % | 56 % | 59 % |
|  |  | Balanced accuracy | 52 % | 52 % | 53 % | 54 % |
|  |  | auROC (95%CI) | 68.70% (68.69-68.70) | 68 % | 69 % | 68 % |
|  |  | auPR (95%CI) | 35.38% (35.37-35.38) | 38 % | 41 % | 43 % |
|  | **MNB** | Sensitivity | 15 % | 16 % | 17 % | 19 % |
|  |  | Specificity | 92 % | 92 % | 91 % | 91 % |
|  |  | Precision | 33 % | 35 % | 39 % | 43 % |
|  |  | Balanced accuracy | 54 % | 54 % | 54 % | 55 % |
|  |  | auROC (95%CI) | 65.84% (65.83-65.85) | 65 % | 66 % | 65 % |
|  |  | auPR (95%CI) | 29.17% (29.16-29.18) | 31 % | 34 % | 38 % |
|  | **NN** | Sensitivity | 5 % | 4 % | 7 % | 13 % |
|  |  | Specificity | 99 % | 99 % | 98 % | 96 % |
|  |  | Precision | 53 % | 57 % | 57 % | 52 % |
|  |  | Balanced accuracy | 52 % | 52 % | 52 % | 54 % |
|  |  | auROC (95%CI) | 68.77% (68.75-68.79) | 68 % | 68 % | 68 % |
|  |  | auPR (95%CI) | 35.25% (35.21-35.28) | 38 % | 40 % | 42 % |
|  | **RF** | Sensitivity | 10 % | 11 % | 13 % | 15 % |
|  |  | Specificity | 96 % | 95 % | 94 % | 93 % |
|  |  | Precision | 36 % | 41 % | 42 % | 42 % |
|  |  | Balanced accuracy | 53 % | 53 % | 54 % | 54 % |
|  |  | auROC (95%CI) | 64.32% (64.29-64.35) | 64 % | 64 % | 63 % |
|  |  | auPR (95%CI) | 29.02% (28.99-29.04) | 31 % | 35 % | 37 % |
|  | **SVM*** | Sensitivity | 1 % | 1 % | 1 % | 1 % |
|  |  | Specificity | 100 % | 100 % | 100 % | 100 % |
|  |  | Precision | 56 % | 55 % | 64 % | 49 % |
|  |  | Balanced accuracy | 51 % | 50 % | 50 % | 50 % |
| **b)** |  |  | **Study Group 1** | **Study Group 2** | **Study Group 3** | **Study Group 4** |
| **SB** | **LR** | Sensitivity | 90 % | 87 % | 83 % | 77 % |
|  |  | Specificity | 24 % | 28 % | 34 % | 42 % |
|  |  | Precision | 67 % | 66 % | 64 % | 63 % |
|  |  | Balanced accuracy | 57 % | 58 % | 59 % | 59 % |
|  |  | auROC (95%CI) | 65.15% (65.14-65.15) | 65 % | 64 % | 65 % |
|  |  | auPR (95%CI) | 74.72% (74.71-74.72) | 73 % | 70 % | 68 % |
|  | **MNB** | Sensitivity | 87 % | 85 % | 83 % | 79 % |
|  |  | Specificity | 23 % | 26 % | 29 % | 33 % |
|  |  | Precision | 66 % | 64 % | 62 % | 60 % |
|  |  | Balanced accuracy | 55 % | 55 % | 56 % | 56 % |
|  |  | auROC (95%CI) | 62.38% (62.36-62.40) | 62 % | 62 % | 61 % |
|  |  | auPR (95%CI) | 73.02% (73.00-73.04) | 71 % | 69 % | 66 % |
|  | **NN** | Sensitivity | 91 % | 87 % | 88 % | 79 % |
|  |  | Specificity | 22 % | 28 % | 25 % | 40 % |
|  |  | Precision | 67 % | 65 % | 62 % | 62 % |
|  |  | Balanced accuracy | 57 % | 58 % | 57 % | 59 % |
|  |  | auROC (95%CI) | 64.84% (64.82-64.86) | 64 % | 64 % | 64 % |
|  |  | auPR (95%CI) | 74.38% (74.36-74.40) | 72 % | 70 % | 68 % |
|  | **RF** | Sensitivity | 80 % | 77 % | 73 % | 68 % |
|  |  | Specificity | 33 % | 36 % | 41 % | 47 % |
|  |  | Precision | 67 % | 65 % | 63 % | 62 % |
|  |  | Balanced accuracy | 56 % | 56 % | 57 % | 57 % |
|  |  | auROC (95%CI) | 60.16% (60.13-60.18) | 60 % | 60 % | 60 % |
|  |  | auPR (95%CI) | 70.40% (70.37-70.43) | 68 % | 66 % | 64 % |
|  | **SVM*** | Sensitivity | 100 % | 100 % | 89 % | 79 % |
|  |  | Specificity | 1 % | 1 % | 25 % | 39 % |
|  |  | Precision | 63 % | 61 % | 63 % | 62 % |
|  |  | Balanced accuracy | 50 % | 50 % | 57 % | 59 % |

| **c)** |  |  | **Study Group 1** | **Study Group 2** | **Study Group 3** | **Study Group 4** |
| --- | --- | --- | --- | --- | --- | --- |
| **VE**** | **LR** | Sensitivity | 0 % | 0 % | 0 % | 0 % |
|  |  | Specificity | 100 % | 100 % | 100 % | 100 % |
|  |  | Precision | 0 % | 0 % | 0 % | 0 % |
|  |  | Balanced accuracy | 50 % | 50 % | 50 % | 50 % |
|  |  | auROC | 50 % | 50 % | 50 % | 50 % |
|  |  | auPR | 17 % | 17 % | 18 % | 18 % |
|  | **MNB** | Sensitivity | 1 % | 2 % | 1 % | 2 % |
|  |  | Specificity | 99 % | 99 % | 99 % | 98 % |
|  |  | Precision | 17 % % | 20 % | 20 % | 18 % |
|  |  | Balanced accuracy | 50 % | 50 % | 50 % | 50 % |
|  |  | auROC | 55 % | 56 % | 55 % | 56 % |
|  |  | auPR | 19 % | 20 % | 20 % | 20 % |
|  | **NN** | Sensitivity | 0 % | 0 % | 0 % | 0 % |
|  |  | Specificity | 100 % | 100 % | 100 % | 100 % |
|  |  | Precision | 0 % | 0 % | 0 % | 7 % |
|  |  | Balanced accuracy | 50 % | 50 % | 50 % | 50 % |
|  |  | auROC | 56 % | 56 % | 55 % | 55 % |
|  |  | auPR | 19 % | 20 % | 20 % | 20 % |
|  | **RF** | Sensitivity | 2 % | 2 % | 2 % | 1 % |
|  |  | Specificity | 98 % | 98 % | 98 % | 99 % |
|  |  | Precision | 20 % | 20 % | 23 % | 16 % |
|  |  | Balanced accuracy | 50 % | 50 % | 50 % | 50 % |
|  |  | auROC | 51 % | 52 % | 51 % | 52 % |
|  |  | auPR | 18 % | 18 % | 19 % | 18 % |
|  | **SVM*** | Sensitivity | 0 % | 0 % | 0 % | 0 % |
|  |  | Specificity | 100 % | 100 % | 100 % | 100 % |
|  |  | Precision | 0 % | 0 % | 0 % | 0 % |
|  |  | Balanced accuracy | 50 % | 50 % | 50 % | 50 % |

SG1: induction of labour (IOL) at 41+0- 41+1 and expectant management (EM) > 41+1, SG2: IOL at 41+2-41+3 and EM> 41+3, SG3: IOL 41+4-41+5 and EM > 41+5; SG4: IOL 41+6-42+0 and EM > 42+0. a) Cesarean delivery (CD). b) spontaneous birth (SB). c) Vaginal operative delivery (VE). Sensitivity, specificity and precision are calculated on a 50 % threshold. LR: logistic regression, MNB: mixed naïve bayes, NN: neural network, RF: random forest, SVM: support vector machine. auROC: area under the receiver operating characteristic curve, auPR: area under the precision-recall curve. 95%CI: 95% confidence interval. * no ROC curves or PR curves were calculated for SVM. ** no confidence intervals were calculated for auROC and auPR in VE.

Roc curve PR curve


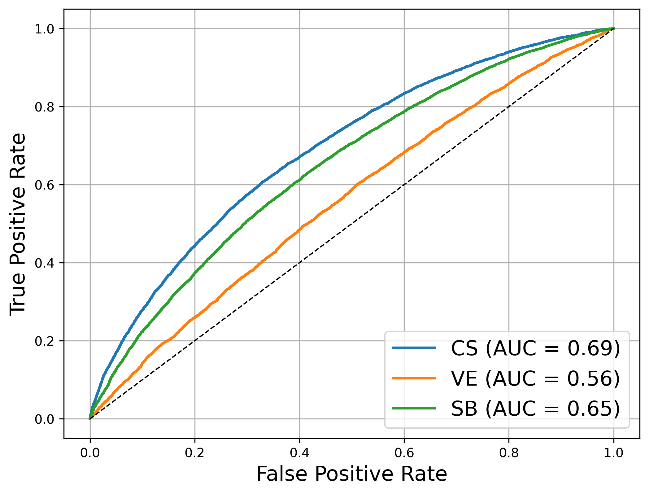

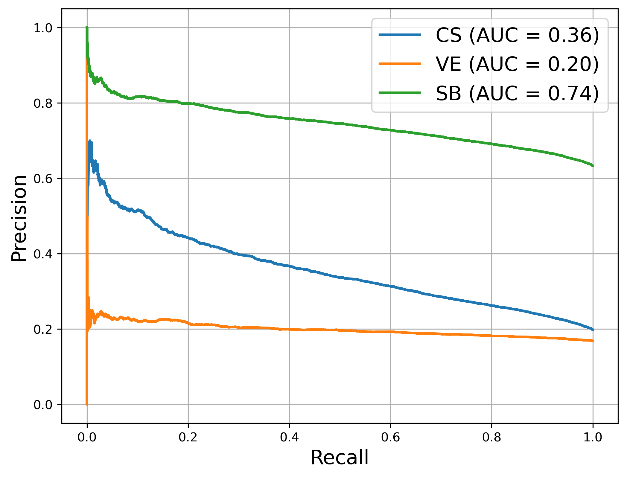


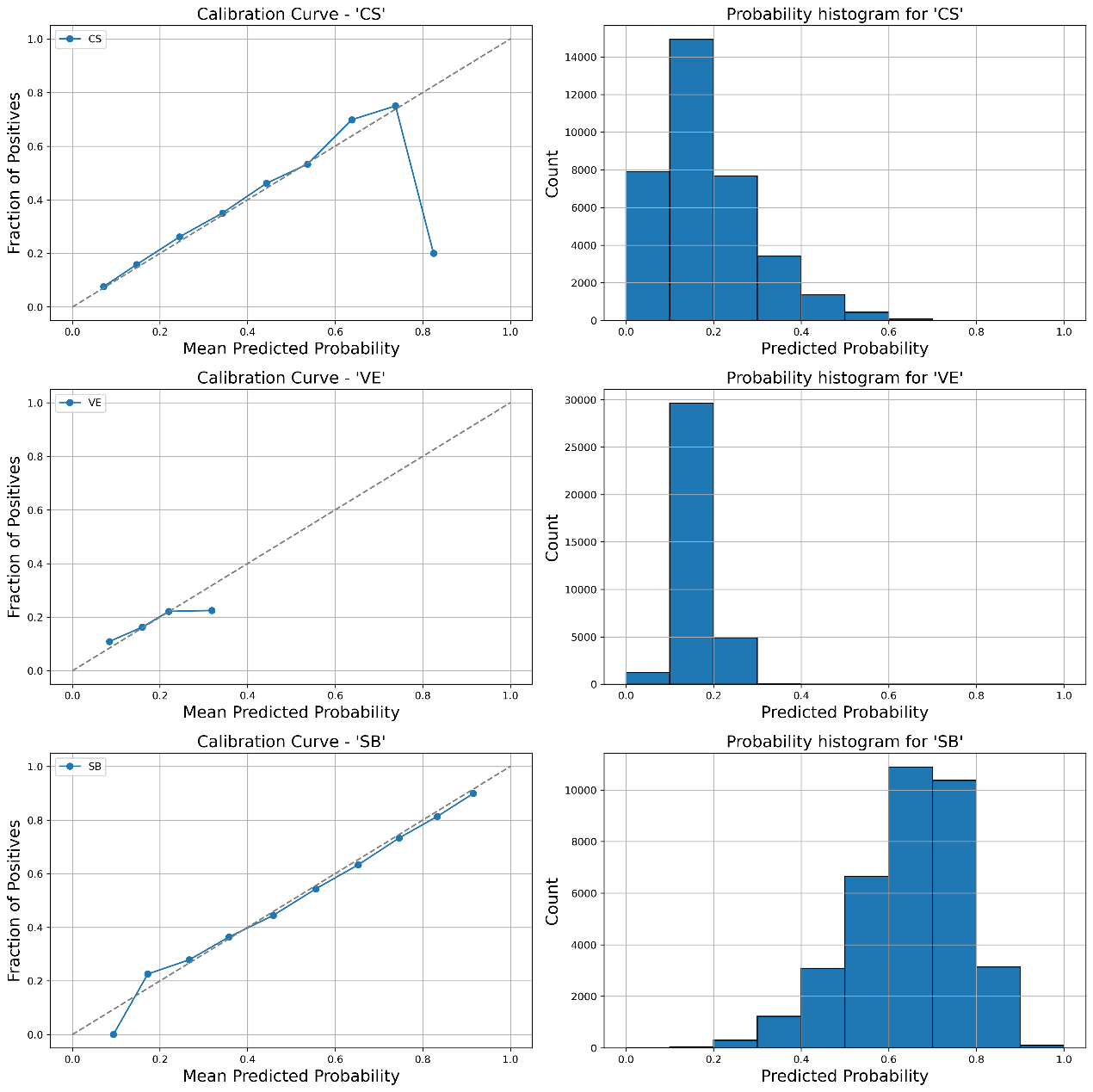


**Figure S10**: Performance metrics (ROC Curves, PR Curves, Calibration Curves) of the models in study group 1 (SG1) in the multiclass prediction with a neural network. ROC curve: receiver operating characteristic curve; PR curve: Precision-Recall Curve; AUC: area under the ROC curve; AP: area under the precision recall curve. CS: cesarean section. SB: spontaneous birth. VE: vacuum extraction. The analysis was run with python version 3.12.7.

Roc curve PR curve


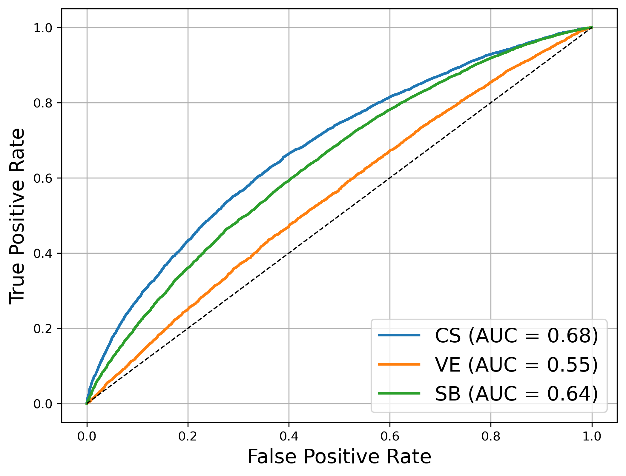

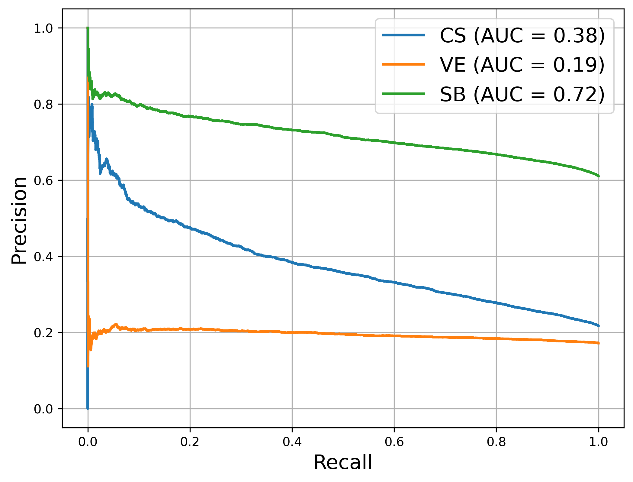


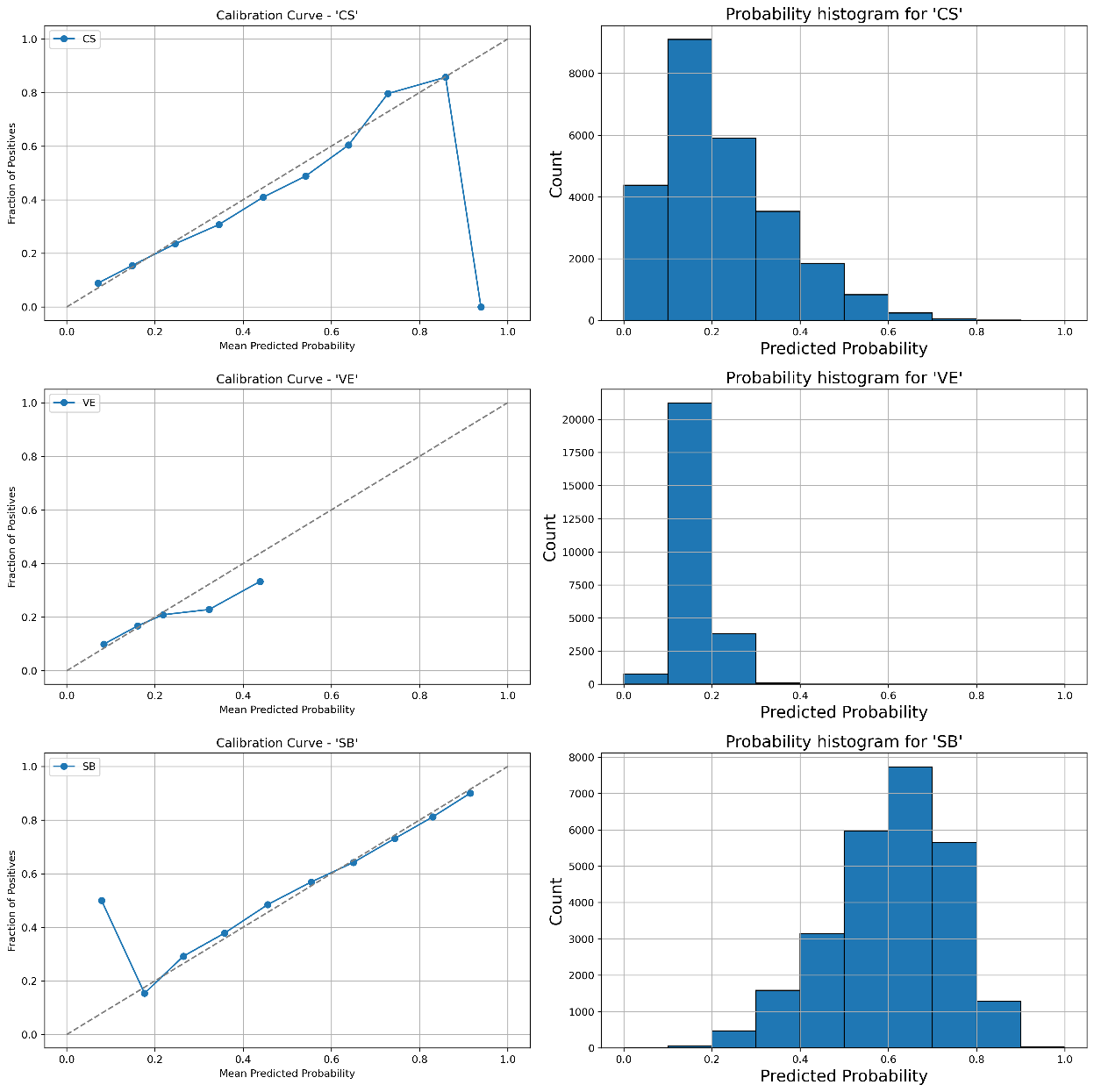


**Figure S11**: Performance metrics (ROC Curves, PR Curves, Calibration Curves) of the models in study group 2 (SG2) in the multiclass prediction with a neural network. ROC curve: receiver operating characteristic curve; PR curve: Precision-Recall Curve; AUC: area under the ROC curve; AP: area under the precision recall curve. CS: cesarean section. SB: spontaneous birth. VE: vacuum extraction. The analysis was run with python version 3.12.7.

Roc curve PR curve


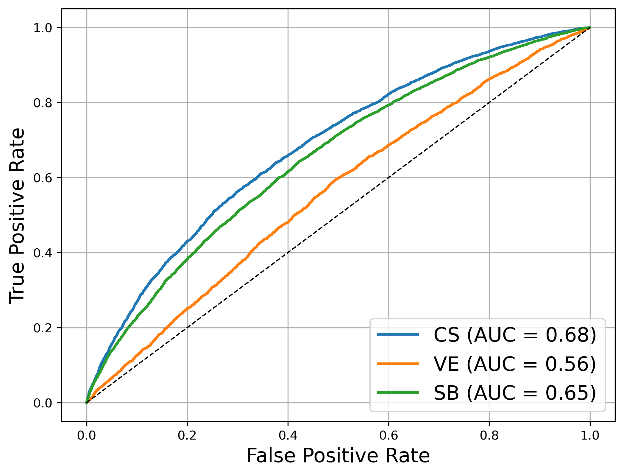

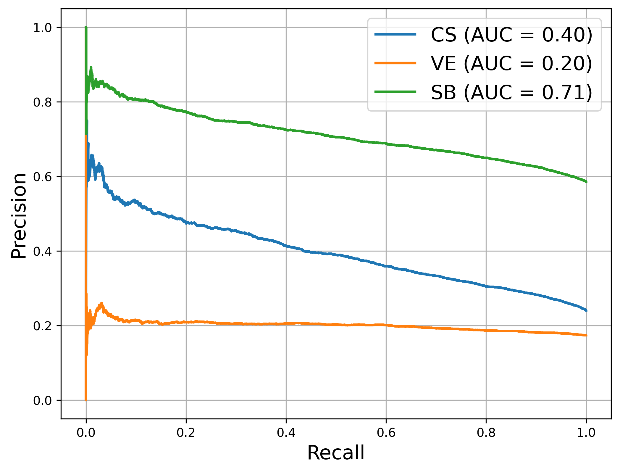


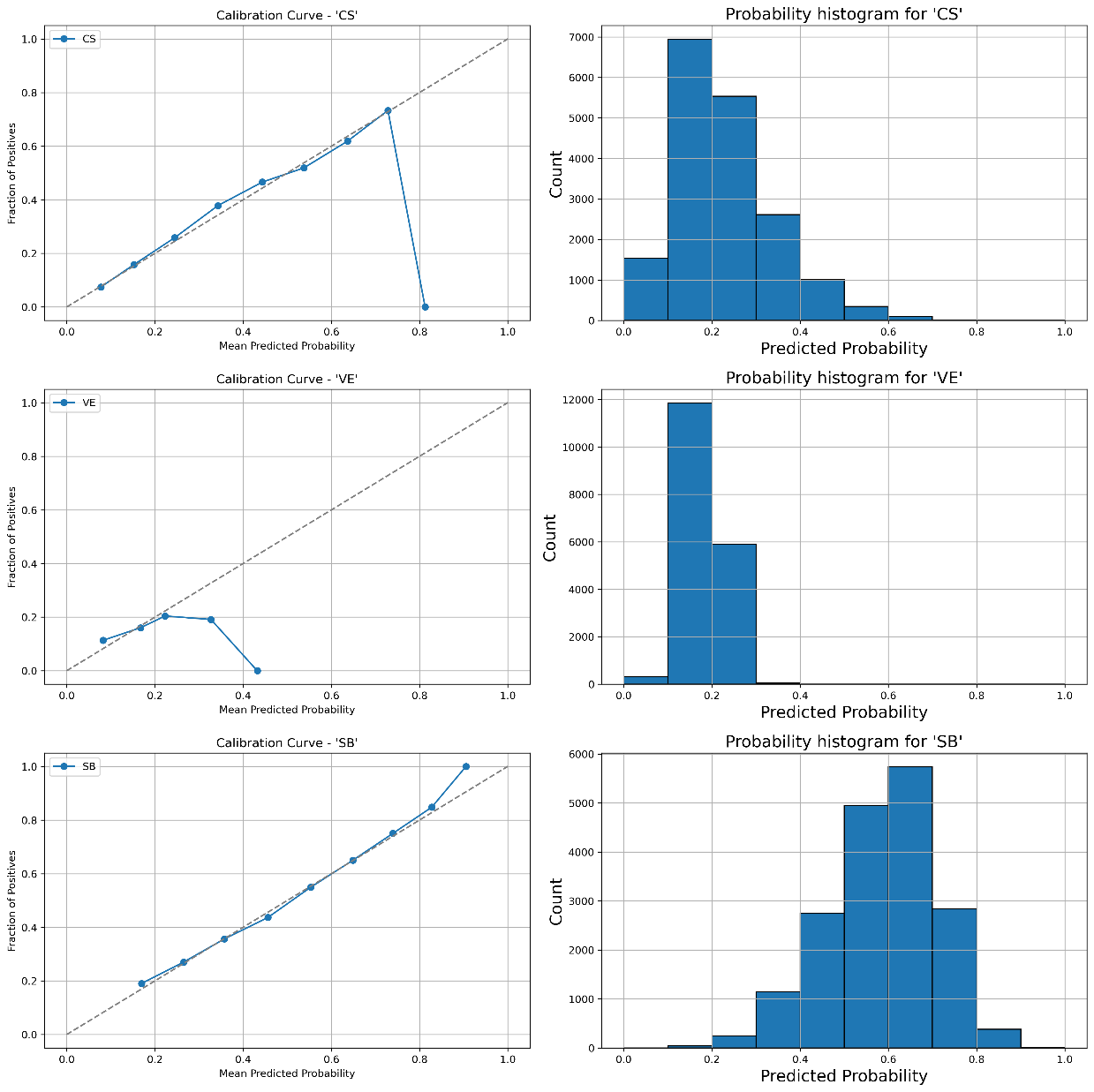


**Figure S12**: Performance metrics (ROC Curves, PR Curves, Calibration Curves) of the models in study group 3 (SG3) in the multiclass prediction with a neural network. ROC curve: receiver operating characteristic curve; PR curve: Precision-Recall Curve; AUC: area under the ROC curve; AP: area under the precision recall curve. CS: cesarean section. SB: spontaneous birth. VE: vacuum extraction. The analysis was run with python version 3.12.7.

Roc curve PR curve


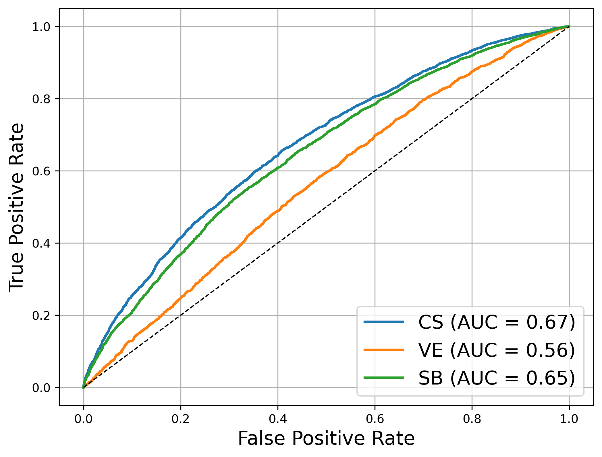

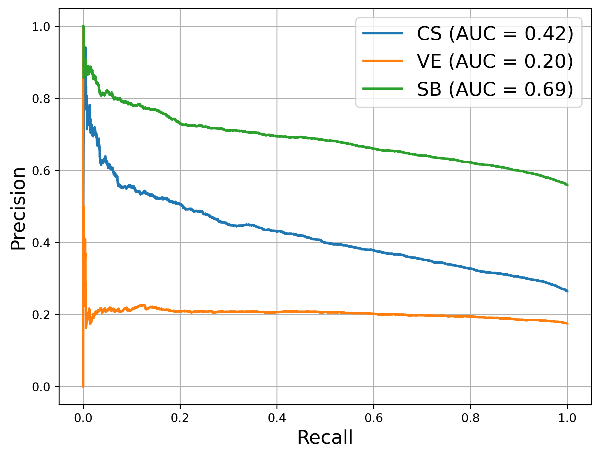


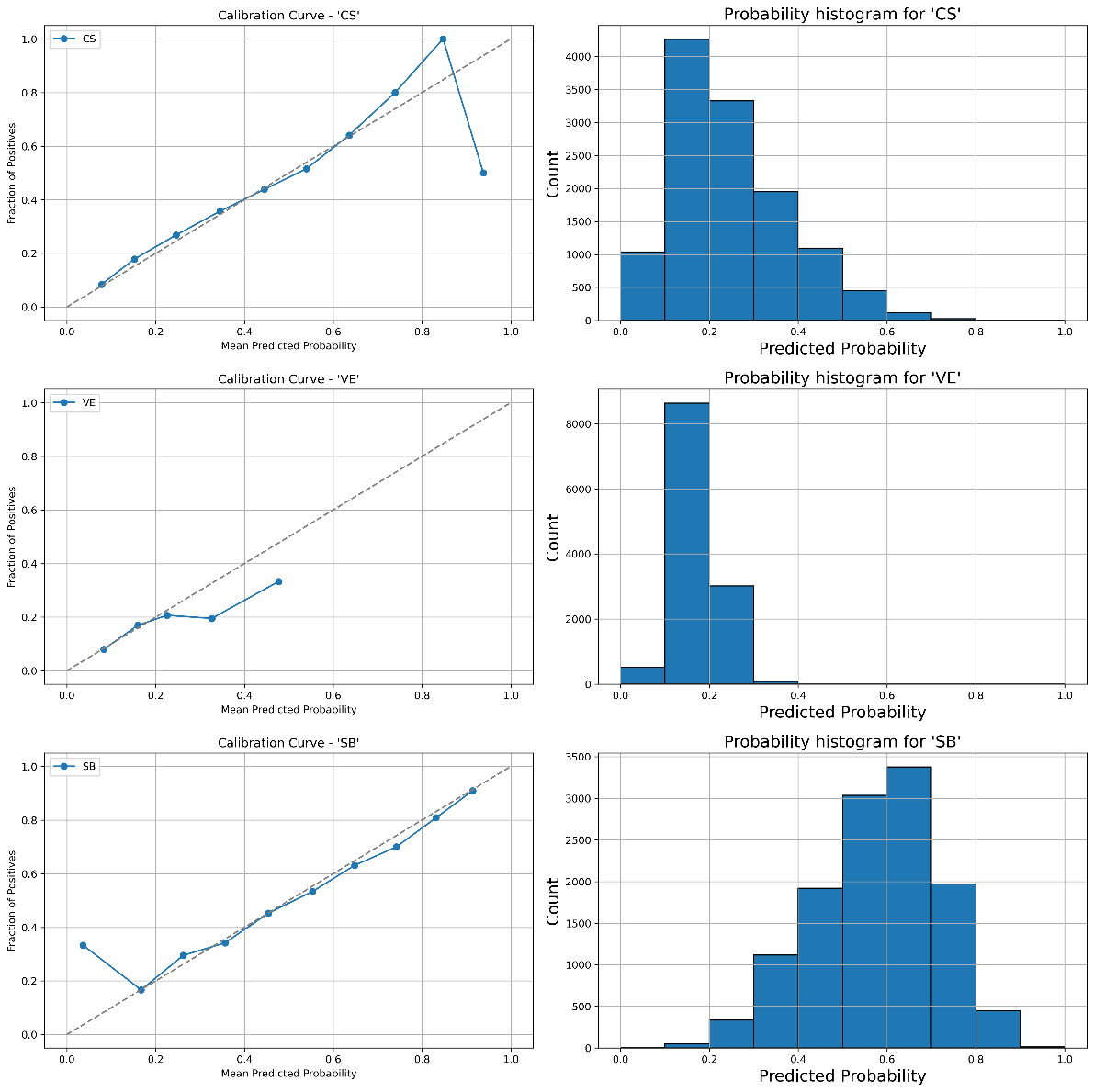


**Figure S13**: Performance metrics (ROC Curves, PR Curves, Calibration Curves) of the models in study group 4 (SG4) in the multiclass prediction with a neural network. ROC curve: receiver operating characteristic curve; PR curve: Precision-Recall Curve; AUC: area under the ROC curve; AP: area under the precision recall curve. CS: cesarean section. SB: spontaneous birth. VE: vacuum extraction. The analysis was run with python version 3.12.7.

**Table S5**: Performance metrics of the different models in Study Group 1-4 (SG1-SG4) for all outcomes in the multiclass prediction with a neural network.

| **performance metrics** | **mode of delivery** | **study group 1** | **study group 2** | **study group 3** | **study group 4** |
| --- | --- | --- | --- | --- | --- |
| Sensitivity | SB | 97% | 94% | 95% | 92% |
|  | VE | 0% | 0% | 0% | 0% |
|  | CD | 12% | 21% | 16% | 23% |
| Precision | SB | 65% | 64% | 61% | 59% |
|  | VE | 0% | 33% | 0% | 50% |
|  | CD | 50% | 47% | 49% | 49% |
| Balanced accuracy |  | 37% | 37% | 37% | 39% |
| auROC | SB | 65% | 64% | 65% | 65% |
|  | VE | 56% | 55% | 56% | 56% |
|  | CD | 69% | 68% | 68% | 67% |
| auPR | SB | 74% | 72% | 71% | 69% |
|  | VE | 20% | 19% | 20% | 20% |
|  | CD | 36% | 38% | 40% | 42% |

SG1: induction of labour (IOL) at 41+0- 41+1 and expectant management (EM) > 41+1, SG2: IOL at 41+2-41+3 and EM> 41+3, SG3: IOL 41+4-41+5 and EM > 41+5; SG4: IOL 41+6-42+0 and EM > 42+0. CD: Cesarean delivery. SB: spontaneous birth. VE: Vaginal operative delivery. auROC: area under the receiver operating characteristic curve, auPR: area under the precision-recall curve.

**Table S6:** Years of included data sources

| **Register** | **Years** |
| --- | --- |
| **National Board of Health and Welfare (NBHW)** |  |
| Medical Birth Register | 1992-2019 |
| Medical Birth Register, Drugs | 1995-2019 |
| Patient Register, Outpatient care | 1997-2021 |
| Patient Register, Inpatient care (hospital admissions) | 1987-2021 |
| Cause of Death Register | 1991-2021 |
| Prescribed Drug Register | 2005-2021 |
|  |  |
| **Statistics Sweden** |  |
| Population Register, death dates | 1952-2021 |
| Population Register, migration | 1908-2021 |
|  |  |
| **National Diabetes Register (NDR)** |  |
| NDR (via Socialstyrelsen) | 1996-2020 |

**Table S7**: Variables of the MBR used in the analysis and the corresponding source.

| Variable | Source | comment |
| --- | --- | --- |
| **maternal** |  |  |
| Personal Identity Number (PIN) | Antenatal record |  |
| Weight at registration to ANC (measured) | Antenatal record |  |
| Height at registration to ANC | Antenatal record | self-reported at first visit |

| Variable | Source | comment |
| --- | --- | --- |
| **maternal** |  |  |
| Smoking 3 months before pregnancy | Antenatal record, check-box | self-reported at first visit |
| Smoking at registration to ANC | Antenatal record, check-box | self-reported at first visit |
| Smoking at 30-32 gestational weeks | Antenatal record, check-box | Self-reported at 30-32 gestational weeks |
| Snuff use 3 months before pregnancy | Antenatal record, check-box | Self-reported at first antenatal visit |
| Snuff use at registration to ANC | Antenatal record, check-box | Self-reported at first antenatal visit |
| Snuff use at 30-32 gestational weeks | Antenatal record, check-box | Self-reported at 30-32 gestational weeks |
| Cohabitation status | Antenatal record, check-box | Self-reported at first antenatal visit. Cohabitation status is defined as: living with “the father-to-be”, living alone, or other family composition. |
| Infertility, years | Antenatal record |  |
| Infertility, in vitro fertilization (IVF) | Antenatal record, check-box | combined into “any assisted reproduction therapy” |
| Infertility, stimulation of ovulation | Antenatal record, check-box | combined into “any assisted reproduction therapy” |
| Infertility, intracytoplasmic sperm injection (ICSI) | Antenatal record, check-box | combined into “any assisted reproduction therapy” |
| Miscarriages, number | Antenatal record, check-box |  |
| **maternal disease** |  |  |
| Recurrent urinary tract infections | Antenatal record, check-box |  |
| Chronic renal disease | Antenatal record, check-box |  |
| Epilepsy | Antenatal record, check-box |  |
| Asthma | Antenatal record, check-box |  |
| Crohn’s disease or ulcerative colitis | Antenatal record, check-box |  |
| Systemic lupus erythematosus | Antenatal record, check-box |  |
| Number of antenatal visits | Calculated by the NBHW |  |
| Primary (essential) hypertension | Antenatal record, check-box | used for creating the variable pre-existing hypertension including ICD codes |
| **Labour and delivery** |  |  |
| Delivery ward - admission | Obstetrical record |  |
| Maternal weight at delivery ward - admission | Obstetrical record |  |
| Spontaneous onset of labour | Obstetrical record, check-box | used for creating the variable onset of delivery (+ICD) |
| Induction of labour | Obstetrical record, check-box | used for creating the variable onset of delivery (+ICD) |
| Cesarean section before onset of labour | Obstetrical record, check-box | used for creating the variable onset of delivery (+ICD) |

| Variable | Source | comment |
| --- | --- | --- |
| **Labour and delivery** |  |  |
| Forceps used any time during delivery | Calculated by the NBHW | Information is based on check-boxes, diagnoses, and surgery codes. For method of delivery, this forceps variable, and the corresponding variables for VE and CS are recommended to be used. |
| Vaccum extraction used any time during delivery | Calculated by the NBHW |  |
| Any caesarean section | Calculated by the NBHW |  |
| Maternal diagnoses during pregnancy, delivery, and post-partum | Obstetrical record | Since 1999, there are 12 fields for recording of maternal diagnoses |
| **Infant** |  |  |
| Date of Birth, text | Neonatal record |  |
| Singleton or multiple birth | Neonatal record |  |
| Presentation at birth | Neonatal record | From 1999: 1=occiput anterior; 4: occiput posterior; 6=breech or foot; 0=other. Maternal and infant diagnoses may also be used to define presentation at birth. |
| Stillbirth | Neonatal record | 1=antepartal, 2=intrapartal |
| Number of deliveries of each mother | Calculated by the NBHW | Calculation is based on information on number of previous deliveries of each mother (from Statistics Sweden and the MBR) |
| Maternal age (completed years) at delivery | Calculated by the NBHW | Calculation of maternal age in completed years is based on the mother’s birth date (included in the first 6 digits in mother’s PIN) and infant’s birth date |
| Paternal citizenship (free text) | Statistics Sweden |  |
| Mother’s country of birth (free text) | Statistics Sweden |  |
| Birth year, infant | Calculated by the NBHW |  |

**Table S8:** Variables calculated prior to the present study and the corresponding source or calculation

| Variable | Source | comment |
| --- | --- | --- |
| mode of delivery | based on any cesarean section, any forceps, or any vacuum extraction used during birth | hierarchical approach beginning with cesarean section, followed by forceps or vaccum extraction. All other birth are classified as spontaneous vaginal birth |
| gestational length at birth | ultrasound measurement, last menstrual period, gestational age set at delivery | hierarchical approach |
| preexisting hypertension | based on the checkbox for hypertension in the MBR and corresponding ICD-10 codes (Table S1) | see also “Primary (essential) hypertension” in the MBR (Table S5) |
| preeclampsia | ICD-10 codes | Table S1 |
| preterm prelabour rupture of the membranes | ICD-10 codes | Table S1 |
